# AS1411-Induced Lipidomic Alterations and Therapeutic Insights in U-87 Glioblastoma Cells

**DOI:** 10.3390/biom16081091

**Published:** 2026-07-25

**Authors:** Ozan Kılıçkaya, Serap Şahin

**Affiliations:** 1 Department of Pharmaceutical Biotechnology, Faculty of Pharmacy, Afyonkarahisar Health Sciences University, 03030 Afyonkarahisar, Türkiye; 2 Department of Biochemistry, Faculty of Pharmacy, Afyonkarahisar Health Sciences University, 03030 Afyonkarahisar, Türkiye

**Keywords:** glioblastoma, nucleolin, aptamer AS1411, lipidomics, mass spectrometry

## Abstract

Glioblastoma (GBM) is a devastating brain tumor heavily reliant on metabolic reprogramming for survival. The DNA aptamer AS1411 specifically targets cell-surface nucleolin (NCL), a protein overexpressed in GBM; however, its precise metabolic consequences remain largely unexplored. This study investigated the direct impact of AS1411-mediated NCL inhibition on the lipidomic profile of U-87 glioblastoma cells. It was demonstrated that AS1411 treatment induced acute cytotoxicity within 24 h. Subsequently, high-resolution mass spectrometry (MS) lipidomics was utilized to identify the lipidomic rewiring triggered by AS1411. Significant changes, such as the upregulation and/or exclusive emergence of specific long-chain and highly polyunsaturated diacylglycerol (DAG), triacylglycerol (TAG), and glycerophospholipid (GP) species, were determined in the species-level analyses. Additionally, our findings revealed that AS1411 treatment induced substantial alterations that profoundly affected membrane biophysics by modifying lipid saturation and acyl chain lengths. An increase in fully saturated sphingomyelin (SM) and cholesteryl ester (CE) levels was observed, leading to the formation of saturated lipid microdomains (lipid rafts) in endosomal and ER membranes, which causes membrane rigidification and decreased fluidity. Our results also demonstrate that PEs containing long-chain polyunsaturated fatty acids (PUFAs)—the primary substrates for ferroptosis—were upregulated. While AS1411 subjects cancer cells to methuotic vacuolization stress, it simultaneously reduces internal structural membrane fluidity and renders the cells metabolically vulnerable to ferroptosis. In conclusion, these detailed lipidomic results indicate that AS1411 treatment proceeds strictly through targeted remodeling and an adaptive scaffolding response.

## 1. Introduction

Primarily sequestered within the nucleolus, Nucleolin (NCL) is a highly conserved and ubiquitous phosphoprotein that exhibits dynamic distribution across the nucleoplasm and cytoplasm. Its translocation to the plasma membrane in various cell types, particularly in malignancies, represents a crucial factor for therapeutic intervention [1,2,3,4]. While integral to fundamental cellular processes like ribosome biogenesis and nucleic acid metabolism [2,5,6], NCL often transforms into a pathological driver in cancer. Frequently overexpressed in malignant tissues, its aberrant surface localization actively contributes to uncontrolled proliferation, tumor growth, and therapeutic resistance [7,8]. The profound reliance of rapidly dividing cancer cells on NCL’s role in protein synthesis creates a critical vulnerability—an “Achilles’ heel” that can be exploited for anti-cancer strategies [1,8,9].

Glioblastoma (GBM) stands as the most prevalent and devastating primary malignant brain tumor in adults, classified by the World Health Organization as a Grade IV astrocytoma, characterized by a grim prognosis and a median survival of merely 15 months [10,11]. Despite aggressive multimodal regimens involving surgery, radiotherapy, and temozolomide (TMZ) chemotherapy—the Stupp protocol—therapeutic outcomes are severely hampered by near-universal tumor recurrence and the blood–brain barrier (BBB) [12,13,14,15,16]. In this dire clinical landscape, NCL has emerged as a particularly relevant player in GBM pathogenesis. Its expression is significantly upregulated in glioma tissues and correlates directly with the grade of malignancy, identifying NCL as a reactivated oncofetal protein that facilitates tumor survival within the complex brain tumor microenvironment [8,17,18,19].

Aptamers, often described as “chemical antibodies,” are short, single-stranded DNA or RNA oligonucleotides that fold into unique three-dimensional conformations, enabling them to bind with high affinity and specificity to a diverse range of molecular targets, including proteins, small molecules, and even whole cells [20,21,22,23]. These molecules are generated through an *in vitro* selection process termed SELEX (Systematic Evolution of Ligands by Exponential Enrichment) [24]. Aptamers present several compelling advantages over traditional monoclonal antibodies as therapeutic agents: their smaller size typically facilitates better tissue penetration; they generally exhibit lower or no immunogenicity; their chemical synthesis is straightforward, scalable, and devoid of biological contaminants; and they can be readily modified to enhance stability (e.g., resistance to nucleases), improve pharmacokinetic profiles, or conjugate therapeutic payloads or imaging agents [25,26,27]. This inherent “programmability” stemming from their chemical nature and SELEX-driven discovery makes aptamers a highly versatile platform for developing precisely engineered and tailored cancer therapies [20,21,28,29,30].

Among the aptamers developed for cancer therapy, AS1411 (AGRO100) has garnered significant attention as a 26-mer guanine-rich DNA oligonucleotide that forms a stable G-quadruplex (G4) structure essential for its biological activity [31,32]. AS1411 specifically targets cell-surface NCL in numerous tumor types, including GBM, where it stimulates its own NCL-dependent internalization via macropinocytosis—a unique “self-potentiating uptake” mechanism [33,34,35]. Once internalized, AS1411 disrupts NCL-mediated functions, including ribosome biogenesis and the stability of oncogenic mRNAs (e.g., Bcl-2), leading to cell cycle arrest and apoptosis [36,37,38]. Although Phase I and II clinical trials for AML and renal cell carcinoma demonstrated a favorable safety profile, AS1411 showed limited efficacy as a monotherapy [31,39]. This clinical experience underscores the need for strategies to enhance its potency or identify responsive patient populations through mechanistic insights. However, the impact of AS1411-mediated NCL inhibition on the complex lipid metabolic landscape of GBM, a key driver of tumor survival and resistance, remains largely unexplored [32].

Concurrently, GBM cells undergo extensive metabolic reprogramming to satisfy heightened bioenergetic demands, with lipid metabolism serving as a central arena [40,41]. Driven by advanced mass spectrometry, lipidomics reveals multifaceted alterations in GBM, including enhanced de novo lipogenesis, dysregulated cholesterol metabolism, and dynamic shifts in specific structural and signaling lipids (e.g., phosphatidylcholines, sphingolipids) [42,43,44,45,46,47]. These alterations are integral to tumor survival, invasion, and therapy resistance, effectively constituting a “survival toolkit” [48,49].

While the roles of NCL in GBM biology and the NCL-targeting activity of AS1411 are increasingly recognized, and the landscape of lipidomic reprogramming in GBM is well-established, a critical knowledge gap exists regarding the direct and specific impact of NCL modulation by AS1411 on the GBM lipidome. Understanding the precise lipidomic consequences of AS1411 treatment could unveil novel mechanisms underlying its anti-tumor effects and identify new lipid-associated changes or synergistic therapeutic targets. Given NCL’s established influence on endothelial cell metabolism and its broad impact on cellular growth and proliferation, a functional connection between NCL activity and the lipidomic landscape of GBM cells or their supporting vasculature is plausible, yet largely unexplored [17].

This study aims to bridge this gap. We hypothesized that targeting NCL with the aptamer AS1411 would induce significant and discernible alterations in the glioblastoma lipidome, potentially revealing novel facets of AS1411’s anti-tumor activity and highlighting new lipid-based therapeutic vulnerabilities. Therefore, the present study aims to: (1) comprehensively characterize the lipidomic profile of GBM cells following AS1411 treatment; (2) identify specific lipid species that are significantly modulated by AS1411-mediated NCL inhibition; and (3) investigate the functional ramifications of these lipidomic alterations on GBM cell viability and proliferation. Such an approach, leveraging lipidomics as a sensitive tool, may uncover previously unappreciated mechanisms by which AS1411 exerts its therapeutic effects and could pave the way for identifying novel signatures of target engagement or therapeutic efficacy.

## 2. Materials and Methods

### 2.1. AS1411 and the Control Aptamer

The unmodified form of AS1411 (5′-GGTGGTGGTGGTTGTGGTGGTGGTGG-3′) and the control aptamer (CRO) (5′-CCTCCTCCTCCTTCTCCTCCTCCTCC-3′) were purchased from GeneScript (Treubstraat 1, Rijswijk, The Netherlands).

### 2.2. Cell Line and Cell Culture Materials

The human glioblastoma cell line (U-87 MG HTB-14^™^) (bacterial and fungal testing also Mycoplasma contamination were not detected) was purchased from American Type Culture Collection (ATCC, Manassas, VA, USA). Dulbecco’s Modified Eagle Medium (DMEM) (Diagnovum, D843) and heat-inactivated fetal bovine serum (Diagnovum D016) were purchased from Diagnovum (Diagnovum GmbH, Greifswald, Germany). Penicillin/Streptomycin Solution (100 U/mL penicillin, and 100 μg/mL streptomycin) (Gibco 15140-122) was purchased from Gibco (Thermo Fisher Scientific, Waltham, MA, USA). Trypsin-EDTA solution (Multicell 325-542-C1) was purchased from Wisent (Wisent Bioproducts, Saint-Jean-Baptiste, QC, Canada). 3-(4,5-dimethylthiazol-2-yl)-2,5-diphenyltetrazolium bromide (MTT) (M2003) and Dimethylsulfoxide (DMSO) (D-8418) were purchased from Sigma (Sigma Aldrich, Darmstadt, Germany).

### 2.3. Cell Line Culture Conditions

The human glioblastoma (U-87 MG HTB-14^™^) cell line was grown in DMEM supplemented with 10% heat-inactivated fetal bovine serum and 1% Penicillin/Streptomycin Solution.

U-87 cells were rapidly thawed in a water bath, and 1 mL of culture medium (prepared with DMEM + 10% FBS + 1% Penicillin/Streptomycin Solution) was added to the thawed cells. After homogenization by pipetting, the cell suspension was transferred into a centrifuge tube containing 9 mL complete culture medium and centrifuged at 300× *g* for 8 min. The supernatant was discarded and the cell pellet was resuspended with complete medium and transferred into a flask (25 cm^2^) containing 8 mL of fresh complete medium, and cultured in a 37 °C humidified incubator with 5% CO_2_ and 95% air.

When cell confluency reached 80–90%, the culture medium was aspirated. Trypsin-EDTA (0.25%) was then added to the flask to facilitate cell detachment. Subsequently, 10 mL of fresh medium was added to each flask to neutralize the trypsin and resuspend the detached cells. The resulting cell suspension was then passaged into new flasks, and the total volume in each flask was adjusted to 8–10 mL with fresh complete culture medium.

U-87 cells were maintained at Afyonkarahisar Health Sciences University, Faculty of Pharmacy, Afyonkarahisar, Türkiye.

### 2.4. Cell Viability Assay

Cells were propagated by incubation in DMEM supplemented with 10% Fetal Bovine Serum (FBS) and 1% Penicillin/Streptomycin Solution under conditions of 95% humidity, 5% CO_2_, and 37 °C. Once cell confluency reached 80–90%, the cells were passaged and seeded into 96-well plates at a density of 2 × 10^5^ cells/mL (100 µL per well). The plates were then incubated for 24 h to allow cell attachment and growth before AS1411 and CRO application. After this 24 h incubation, cells were treated with varying concentrations (0–80 µM) of AS1411 and CRO dissolved in sterile distilled water (1 µL for per well). In control wells (untreated with AS1411 or CRO) added 1 µL of sterile distilled water (1% final concentration), which served as the solvent for the AS1411 and CRO. The plates were subsequently incubated in a 95% humidity, 5% CO_2_, 37 °C incubator for 24, 48, and 96 h. At the end of each incubation period, 10 µL of MTT (3-(4,5-dimethylthiazol-2-yl)-2,5-diphenyltetrazolium bromide) solution (5 mg/mL in PBS, pH 7.4) was added to each well, and the plates were re-incubated in the CO_2_ incubator for an additional 3 h. Following this, the medium in the plates was aspirated, and 100 µL of DMSO was added to each well. The plates were then incubated on a shaker at room temperature for 15 min to dissolve the formazan crystals. Sample absorbances were measured at 570 nm using an ELISA plate reader (Bio Tek EPOCH, Santa Clara, CA, USA) [50]. Cell viability was calculated using the following formula:

% Cell Viability = (Sample Absorbance/Control Absorbance) × 100

### 2.5. Lipid Extraction for Mass Spectrometry Lipidomics

Mass spectrometry-based lipid analysis was performed by Lipotype GmbH (Dresden, Germany) as described in [51]. Cells were incubated with a clinically achievable concentration of AS1411 (5 µM) for 24 h [39]. Lipids were extracted using a two-step chloroform/methanol procedure, with chloroform:methanol 17:1 (V:V) and 2:1 (V:V) in the first and second step, respectively [52]. Prior to extraction, 20 µL samples (Tables S1 and S2) were spiked with internal lipid standard mixture containing: 100 pmol of cholesterol ester 16:0 D7 (CE), 100 pmol of diacylglycerol 17:0/17:0 (DAG), 50 pmol of phosphatic acid (PA) 17:0/17:0 (PA), 500 pmol of phosphatidylcholine 15:0/18:1 D7 (PC), 50 pmol of phosphatidylethanolamine 17:0/17:0 (PE), 50 pmol of phosphatidylglycerol 17:0/17:0 (PG), 50 pmol of phosphatidylinositol 16:0/16:0 (PI), 50 pmol of phosphatidylserine (PS) 17:0/17:0 (PS), 200 pmol of sphingomyelin (SM) 18:1;2/12:0;0 (SM) and 50 pmol of triacylglycerol 17:0/17:0/17:0 (TAG). After extraction, the organic phase was transferred to an infusion plate and dried in a speed vacuum concentrator. The dry extract was re-suspended in 7.5 mM ammonium formiate in chloroform/methanol/propanol (1:2:4; V:V:V). All liquid handling steps were performed using Hamilton Robotics STARlet robotic platform with the Anti Droplet Control feature for organic solvents pipetting [53].

### 2.6. MS Data Acquisition

Samples were analyzed by direct infusion on a QExactive mass spectrometer (Thermo Scientific, San Jose, CA, USA) equipped with a TriVersa NanoMate ion source (Advion Biosciences, Ithaca, NY, USA). Samples were analyzed in both positive and negative ion modes with a resolution of R_*m*/*z*=200_ = 280,000 for MS and R_*m*/*z*=200_ = 17,500 for MSMS experiments, in a single acquisition. MSMS was triggered by an inclusion list encompassing corresponding MS mass ranges scanned in 1 Da increments [53]. Both MS and MSMS data were combined to monitor CE [colesterol ester acetate + NH4]^+^ *m*/*z* 369.352, DAG [DAG acetate + NH4]^+^ *m*/*z* 17.023, and TAG [TAG acetate + NH4]^+^ *m*/*z* 17.023, ions as ammonium adducts, PC [PC formiate + HCOO]^−^ *m*/*z* 60.021, as formiate adducts, and PA [PA-H]^−^ *m*/*z* 152.9958, PE [PE-H]^−^ *m*/*z* 140.012, PG [PG-H]^−^ *m*/*z* 152.996, PI [PI-H]^−^ *m*/*z* 241.012, and PS [PS-H]^−^ *m*/*z* 87.032 as deprotonated anions. MS was only used to monitor SM as formiate adducts.

### 2.7. Data Analysis and Post-Processing

Data were analyzed with in-house developed lipid identification software based on LipidXplorer [54,55]. Data post-processing and normalization were performed using an in-house-developed data management system. Only lipid identifications with a signal-to-noise ratio > 5, and a signal intensity 5-fold higher than in corresponding blank samples were considered for further data analysis. Only lipids with amounts pmol and identified in at least three out of five biological replicates are reported.

### 2.8. Statistical Analysis

Statistical analyses were performed using GraphPad Prism 8 (GraphPad Software, San Diego, CA, USA). Unless otherwise specified in the figure legends, all data are presented as mean ± standard deviation (SD) (*n* = 5). Group comparisons were conducted using either Student’s *t*-test (two groups) or one-way ANOVA (>two groups), followed by Tukey’s post hoc test for multiple comparisons. Significance was set at *p* < 0.05 (* for *p* < 0.05 vs. Control; # for *p* < 0.05 vs. CRO).

## 3. Results

### 3.1. Cytotoxicity of AS1411 and CRO Against U-87 Cells

**Figure 1:**
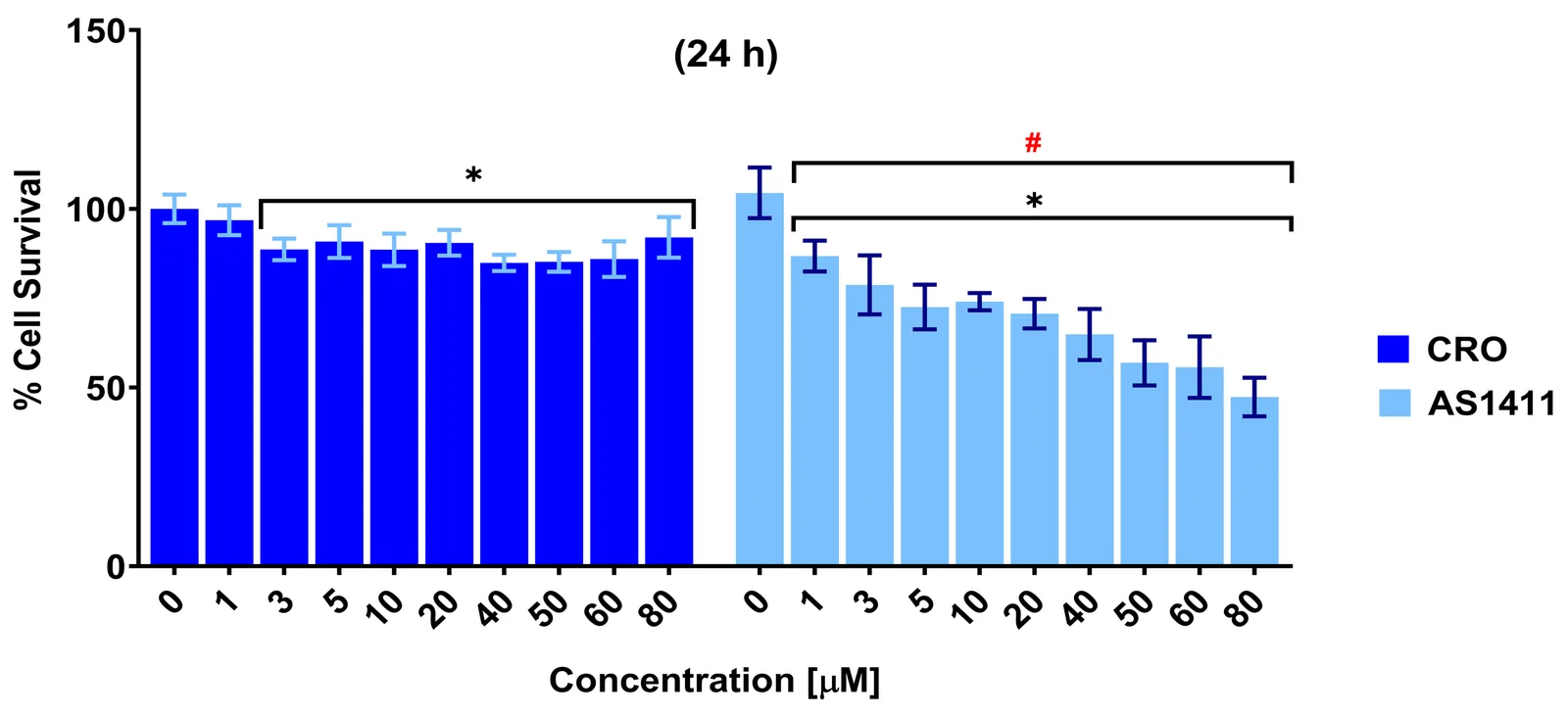
Cell viability assessed by MTT assay following 24 h treatment with varying concentrations of AS1411 and CRO against U-87 cells. Statistical significance: * *p* < 0.05 vs. Control; # *p* < 0.05 vs. CRO. CRO: Control aptamer.

To elucidate the molecular mechanisms underlying AS1411’s activity, we first evaluated its direct impact on U-87 glioblastoma cell viability. AS1411 exhibited significant cytotoxicity with distinct dose-dependent activity patterns. MTT viability assays revealed maximum cytotoxic efficacy at 24 h, with AS1411 reducing cell viability in a dose-dependent manner, whereas the CRO showed minimal effects (Figure 1).

Notably, no cytotoxic activity was observed at extended time points of 48 and 96 h (Figures S1 and S2). The CRO remained essentially non-toxic across all time points, confirming specificity. In our cell viability assays, AS1411 concentrations ranging from 3 to 40 µM for 24 h did not exhibit statistically significant differences compared to one another regarding U-87 cell viability. At the clinically relevant dose of 5 µM [39], cell viability was maintained at approximately 75%. Consequently, cells were incubated with this clinically achievable concentration of AS1411 (5 µM) for 24 h for lipidomic analyses.

### 3.2. Targeted Nucleolin Inhibition by AS1411 Substantially Alters the Lipid Profile of U-87 Cells

**Figure 2:**
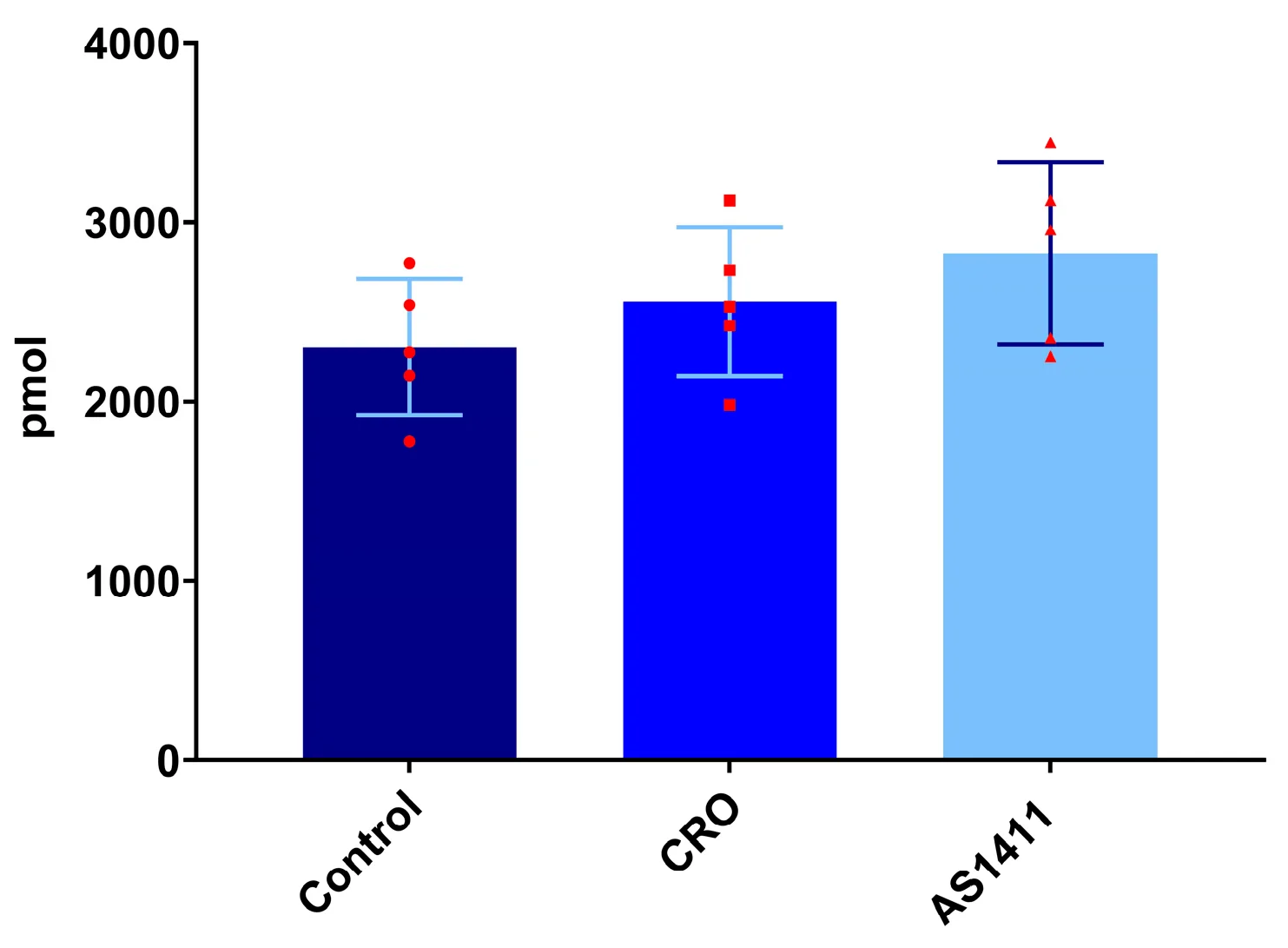
Total lipid measured in samples. Bars represent means ± SD (*n* = 5), with the individual samples shown as points. Control: Untreated cells, CRO: Control aptamer.

When examining the absolute total mass of the cellular lipidome, a gradual upward trend is observed from the Control to the CRO, and subsequently to the AS1411. However, this upward trend is not statistically significant (Figure 2). The analytical reproducibility was assessed by plotting the variation in lipid levels in quality control reference samples (*n* = 2 ±SD), including all lipid species present across any of the replicates (Figure S3; Table S3).

**Figure 3:**
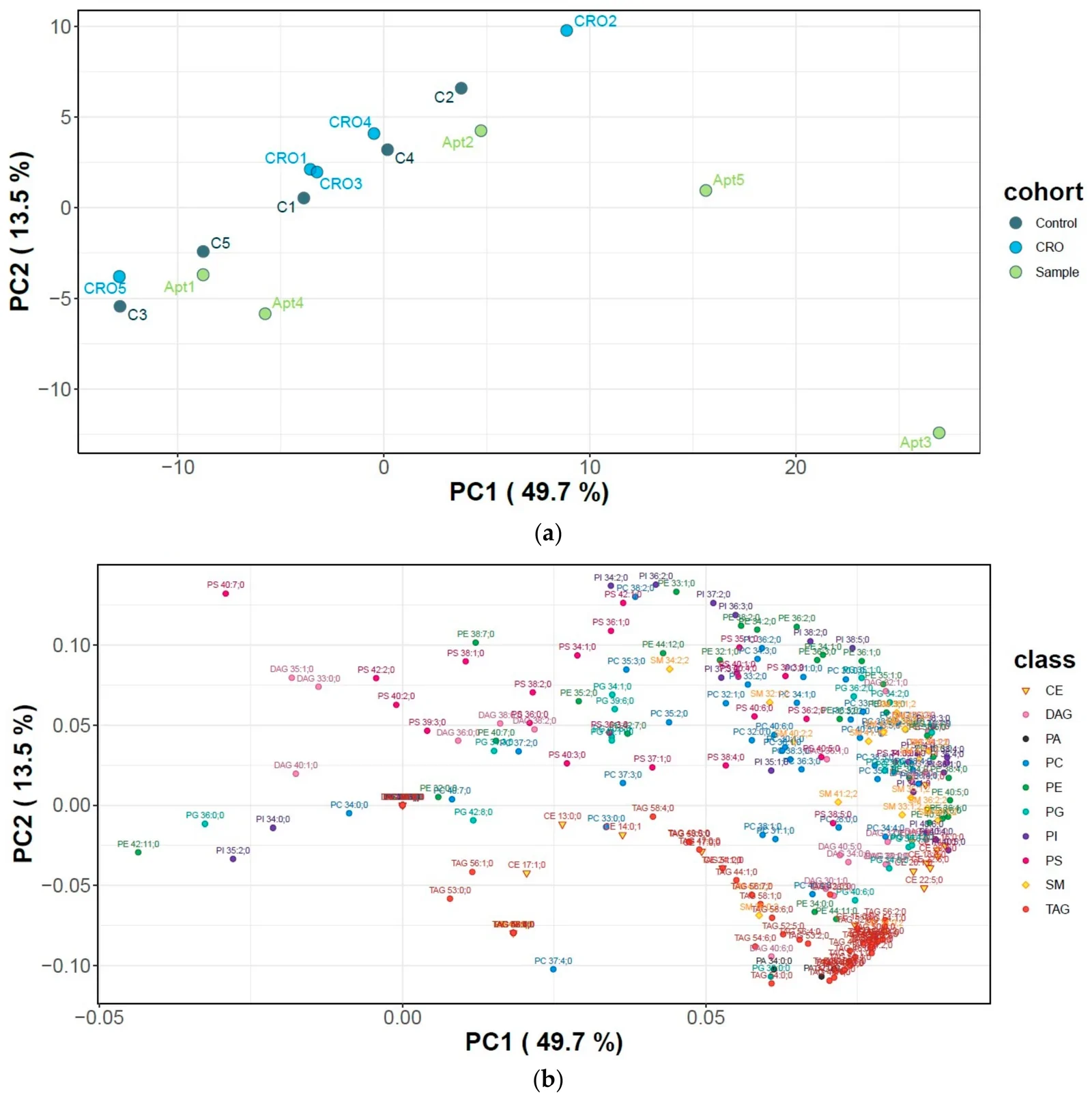
Multivariate analysis of the lipidomic remodeling. PCA (**a**) scores plot and (**b**) loadings plot based on absolute quantification data (pmol), illustrating the segregation of the AS1411-treated group (green) away from Control (untreated, gray) and CRO (blue) groups. CRO: Control aptamer.

**Figure 4:**
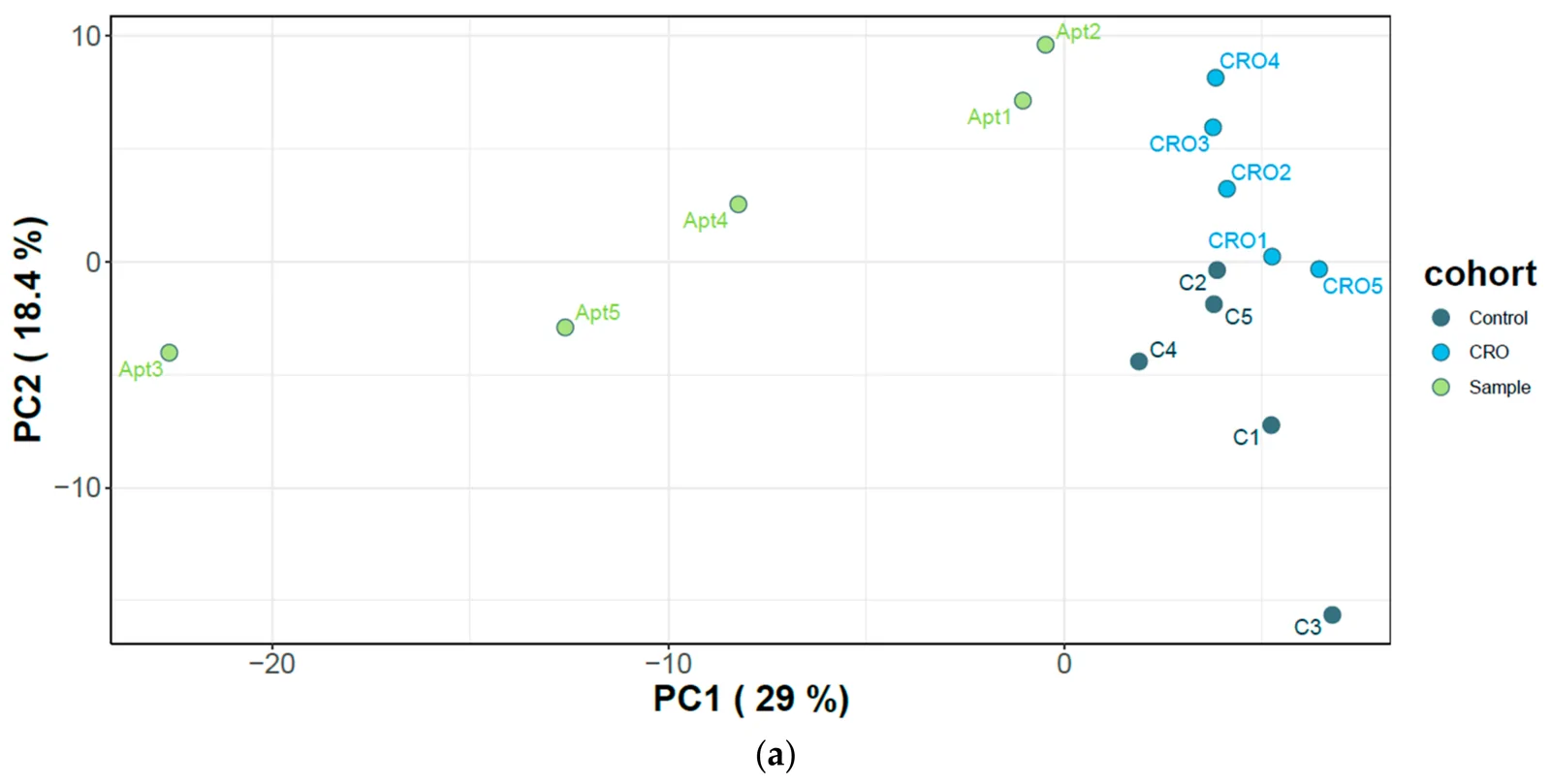

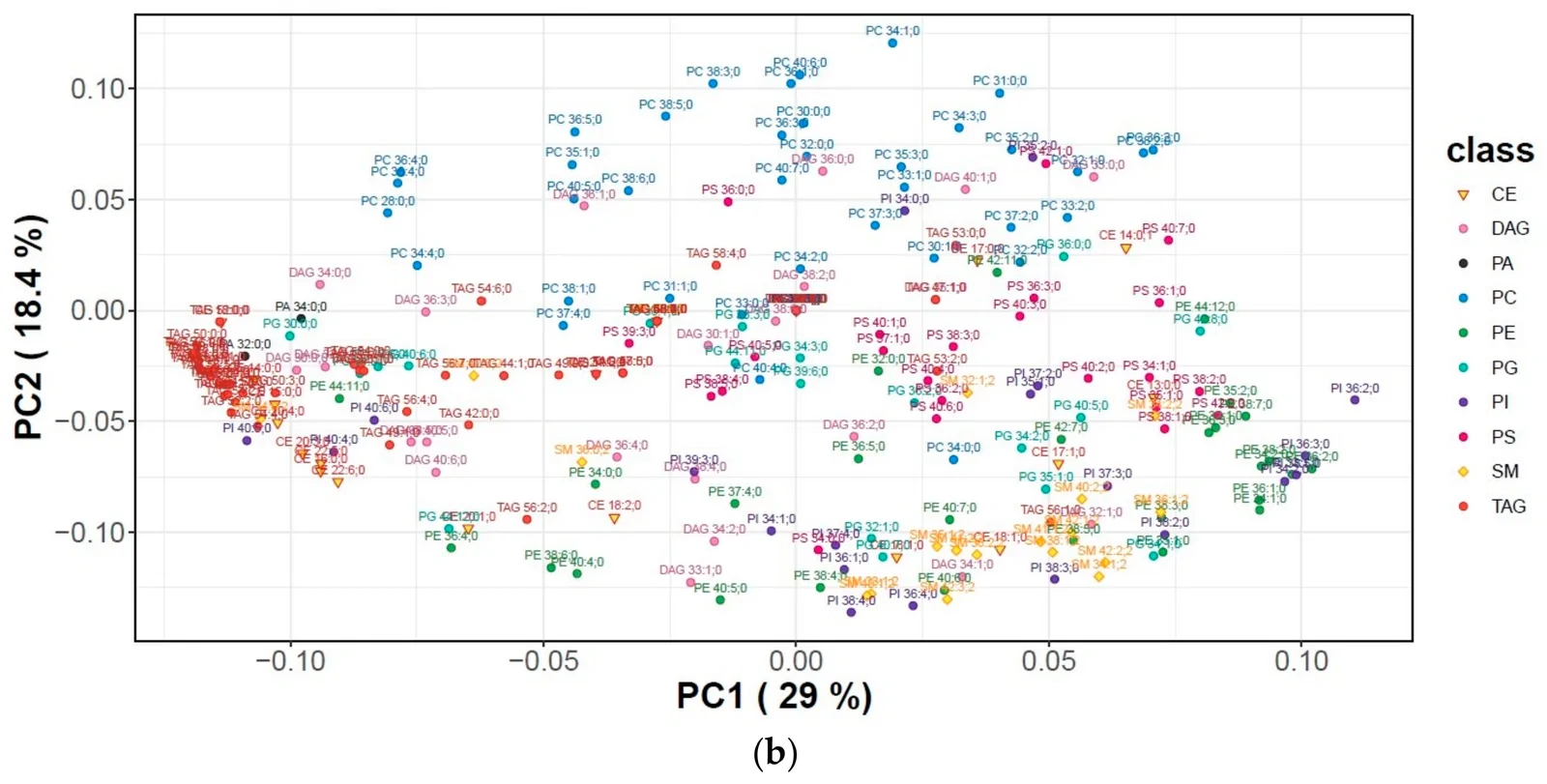
PCA of the lipidomic profiles. (**a**) Scores plot and (**b**) loadings plot generated using relative lipid abundances (mol%), demonstrates segregation of AS1411-treated cells (green) from the Control (gray) and CRO (blue) groups. CRO: Control aptamer.

A clear separation between groups is compelling evidence that the AS1411 induces a specific and potent remodeling of the cellular lipidome. Principal Component Analysis (PCA) findings based on absolute quantification (pmol) data reflect the total lipid mass and biomass expansion within the cell. In the PCA model based on absolute lipid quantities, 63.2% of the total variance is explained by the first two principal components (PC1: 49.7%, PC2: 13.5%) (Figure 3). The PCA model based on relative distribution (mol%) explains 47.4% of the total variance (PC1: 29.0%, PC2: 18.4%) (Figure 4).

**Figure 5:**
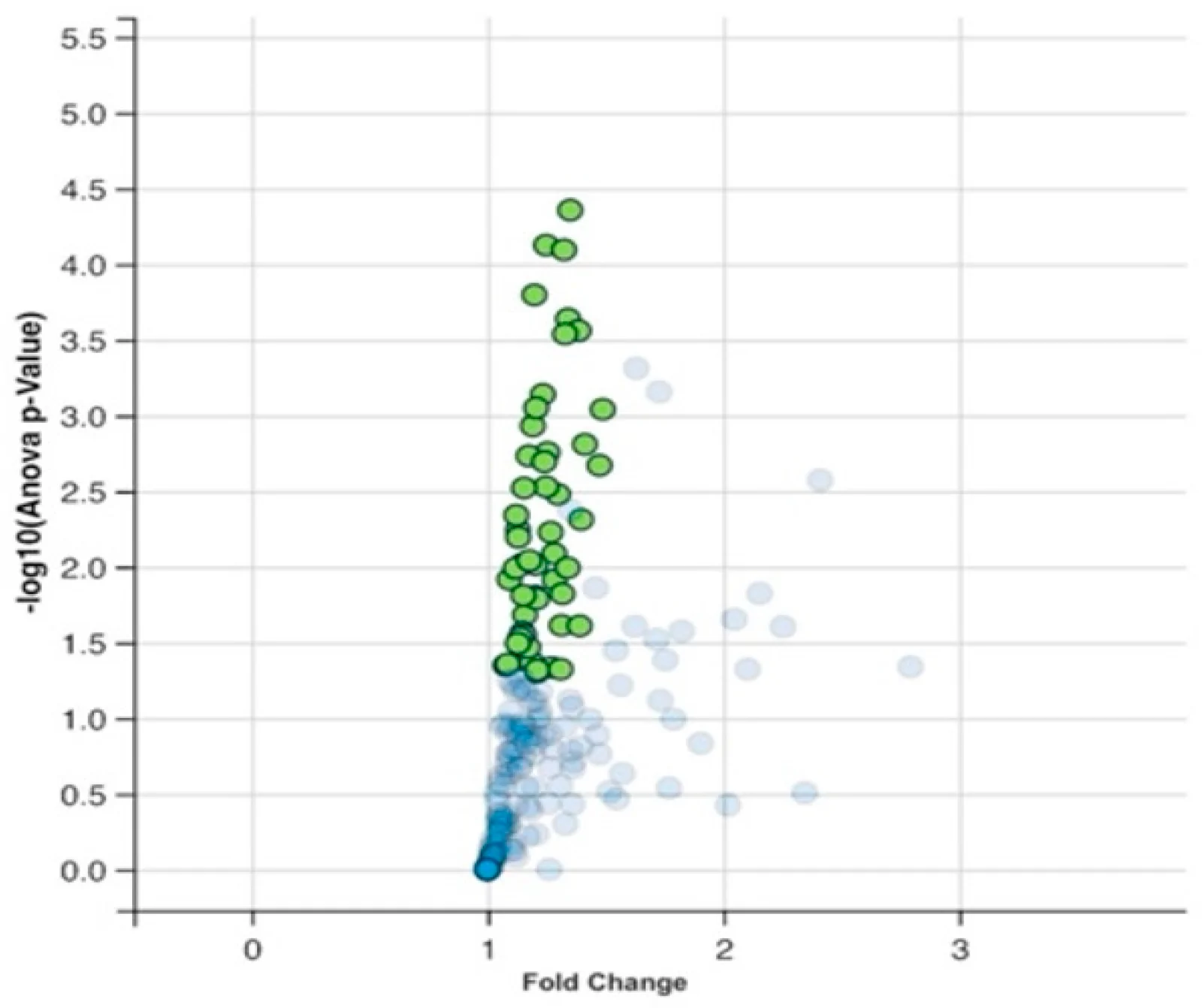
Volcano Plot visualizing the significantly altered lipid species. Green dots indicate upregulated lipids, while blue dots indicate downregulated lipids.

Analyzing the Volcano Plot that maps the impact of AS1411 treatment on the cellular lipidome (Figure 5), two distinct metabolic populations emerge, comprising lipid species that exhibit either an increase or a decrease. Specifically, the lipid species showing an increase following treatment are indicated as green data points, while the decreasing lipid species are indicated as blue data points.

### 3.3. Targeted Alterations in Specific Lipid Classes and Membrane Architecture

Lipid molecules are identified as species or sub-species. Fragmentation of the lipid molecules in MSMS mode delivers sub-species information, i.e., the exact acyl chain (e.g., fatty acid) composition of the lipid molecule. MS only mode, acquiring data without fragmentation, cannot deliver this information and provides species information only. In that case, the sum of the carbon atoms and double bonds in the hydrocarbon moieties is provided. Lipid species are annotated according to their molecular composition as lipid class <sum of carbon atoms>:<sum of double bonds>;<sum of hydroxyl groups>. For example, PI 34:1;0 denotes phosphatidylinositol with a total length of its fatty acids equal to 34 carbon atoms, total number of double bonds in its fatty acids equal to 1 and 0 hydroxylations. In case of sphingolipids, SM 34:1;2 denotes a sphingomyelin species with a total of 34 carbon atoms, 1 double bond, and 2 hydroxyl groups in the ceramide backbone (Table S4).

**Figure 6:**
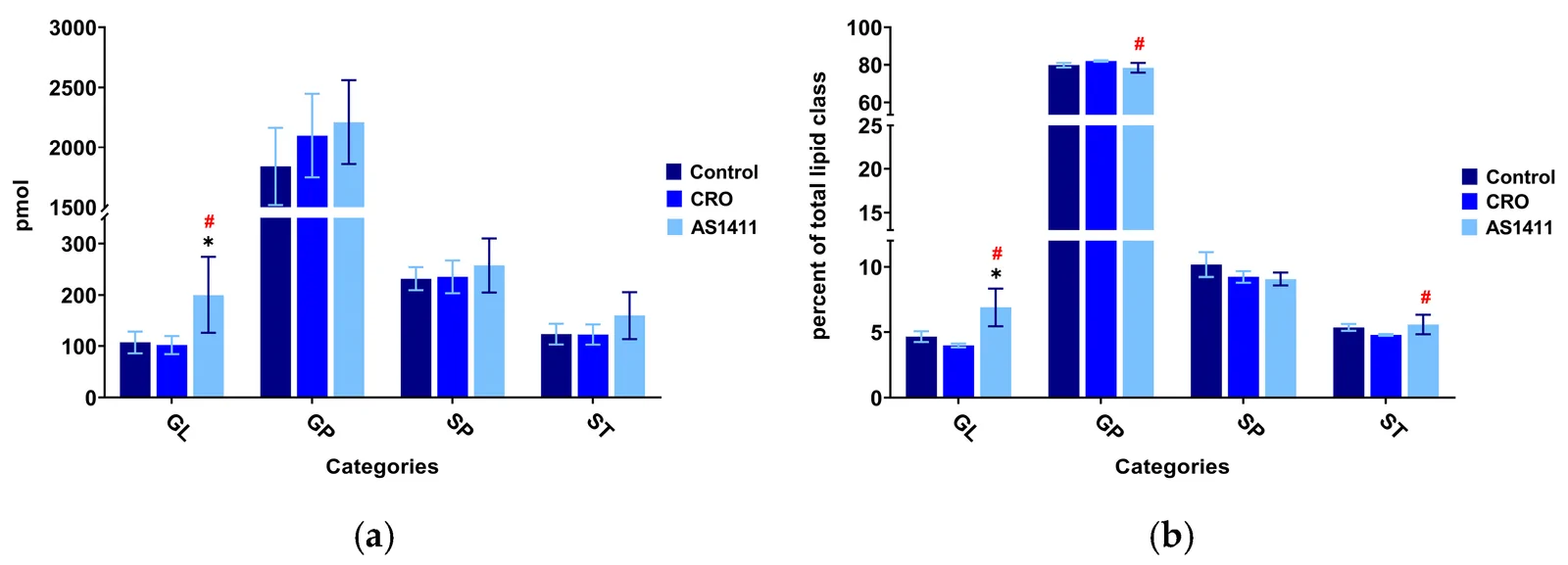
Lipid categories (GL, GP, SP, and ST) in U-87 cells following AS1411 and CRO treatment. (**a**) Absolute quantification of the lipid categories based on molar amounts (pmol). (**b**) Relative abundance of the lipid categories standardized to the total lipid content (mol%). CRO: Control aptamer. * *p* < 0.05 vs. Control; # *p* < 0.05 vs. CRO.

**Figure 7:**
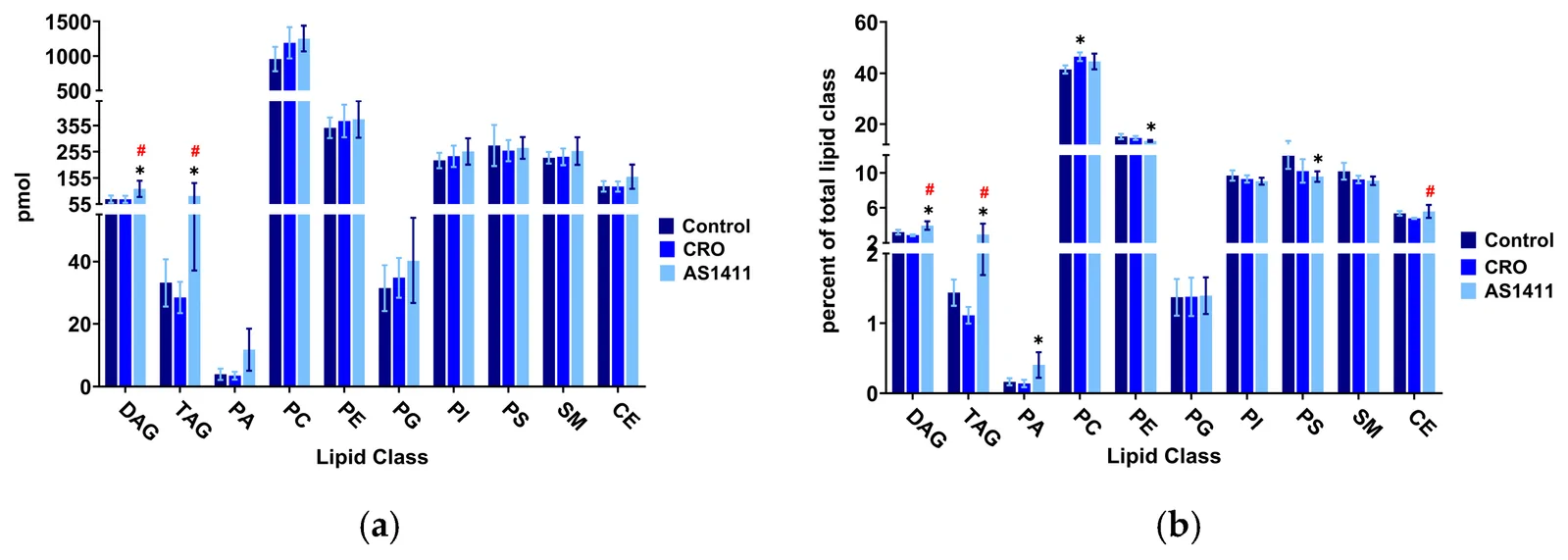
Lipid classes (DAG, TAG, PA, PC, PE, PG, PI, PS, SM, and CE) in U-87 cells following AS1411 and CRO treatment. (**a**) Absolute quantification of the lipid classes based on molar amounts (pmol). (**b**) Relative abundance of the respective lipid classes standardized to the total lipid content (mol%). * *p* < 0.05 vs. Control; # *p* < 0.05 vs. CRO.

Lipidomic profiling reveals a highly selective, sequence-dependent metabolic modulation by the AS1411. The most prominent alteration is observed in the GL category, which demonstrates a sequence-specific expansion in the AS1411-treated group (Figure 6a,b). Conversely, the GP and ST pools exhibit statistical divergence primarily driven by the CRO rather than a direct AS1411 effect (Figure 6b). Further dissection into lipid classes confirms that the GL category expansion is entirely propelled by significant, sequence-specific accumulations in DAG and TAG pools (Figure 7a,b). However, structural phospholipids present a highly variable landscape (Figure 7b).

**Figure 8:**
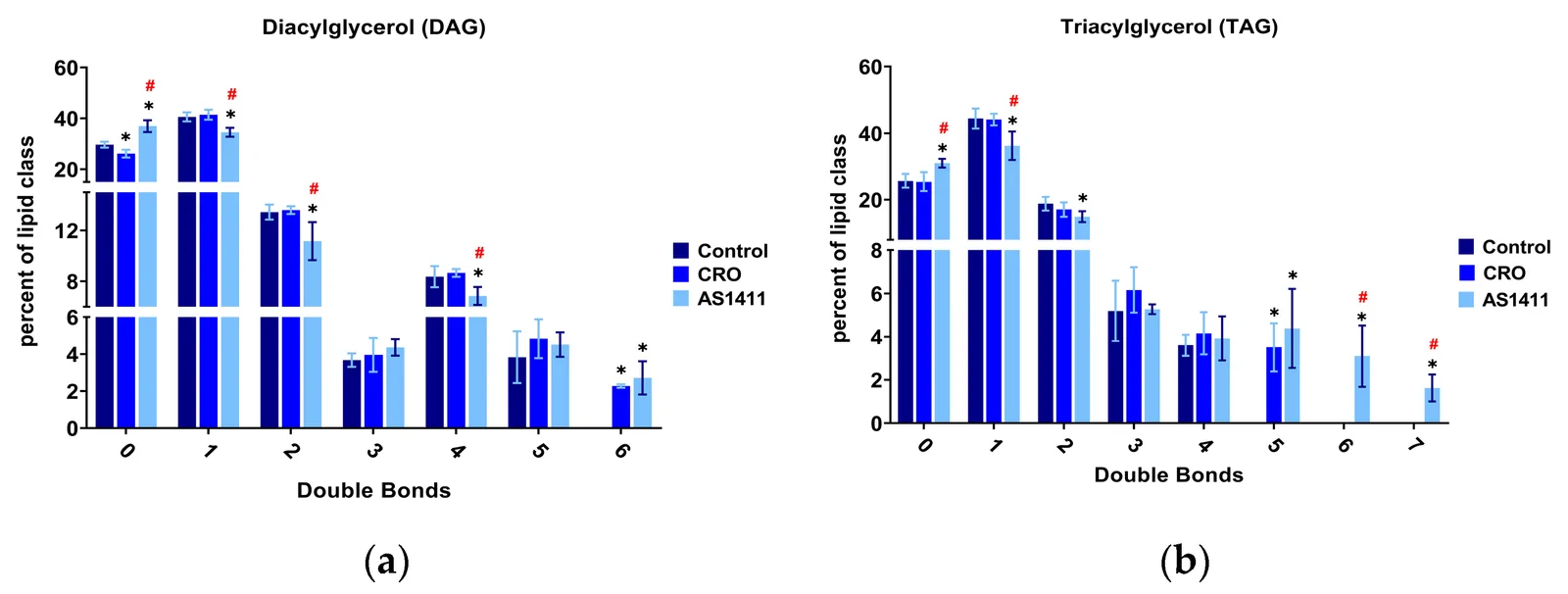
Total double bond profile of GL class including (**a**) DAG, (**b**) TAG in U-87 cells. * *p* < 0.05 vs. Control; # *p* < 0.05 vs. CRO.

**Figure 9:**
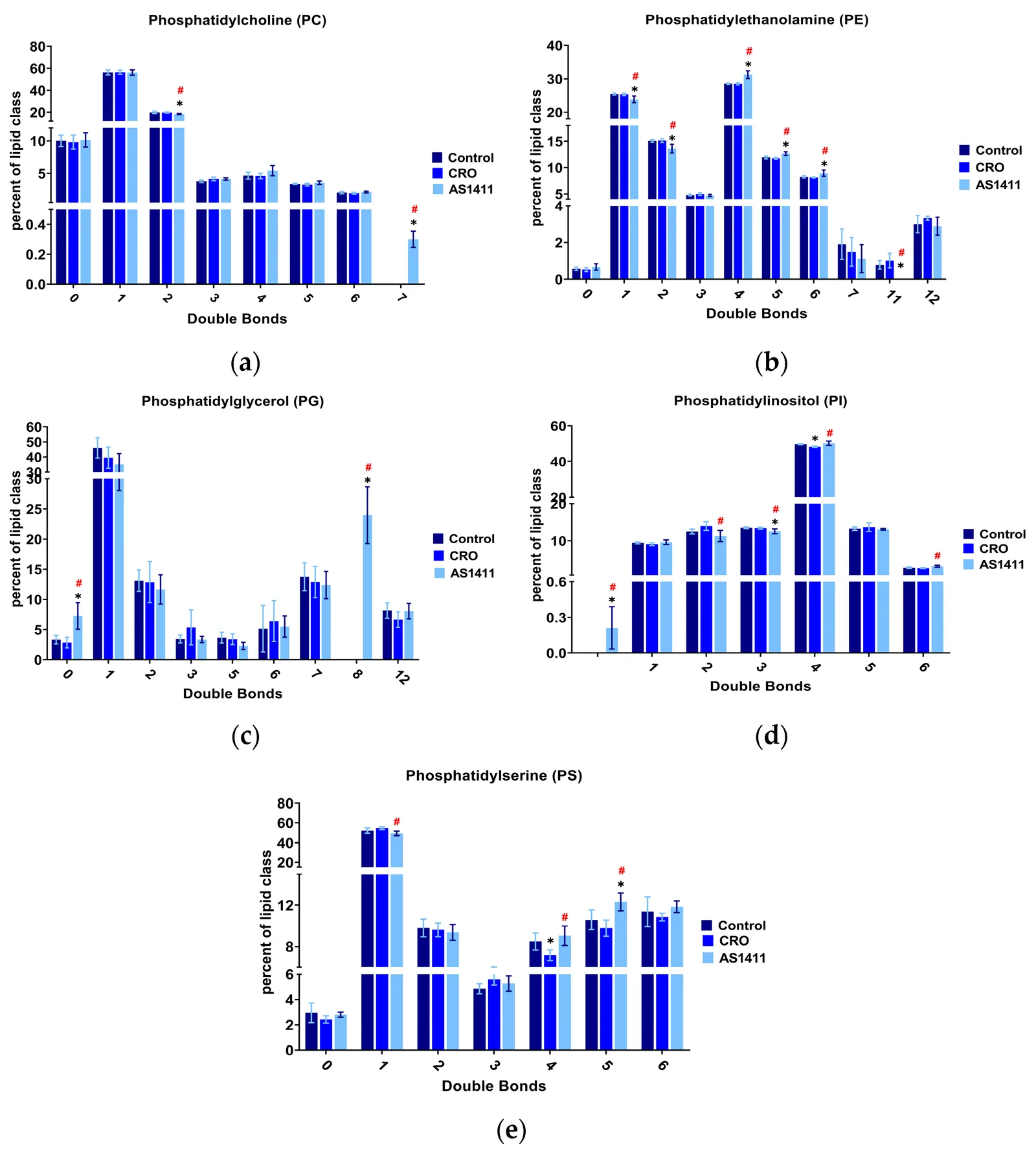
Total double bond profile of GP class including (**a**) PC, (**b**) PE, (**c**) PG, (**d**) PI, and (**e**) PS in U-87 cells. * *p* < 0.05 vs. Control; # *p* < 0.05 vs. CRO.

**Figure 10:**
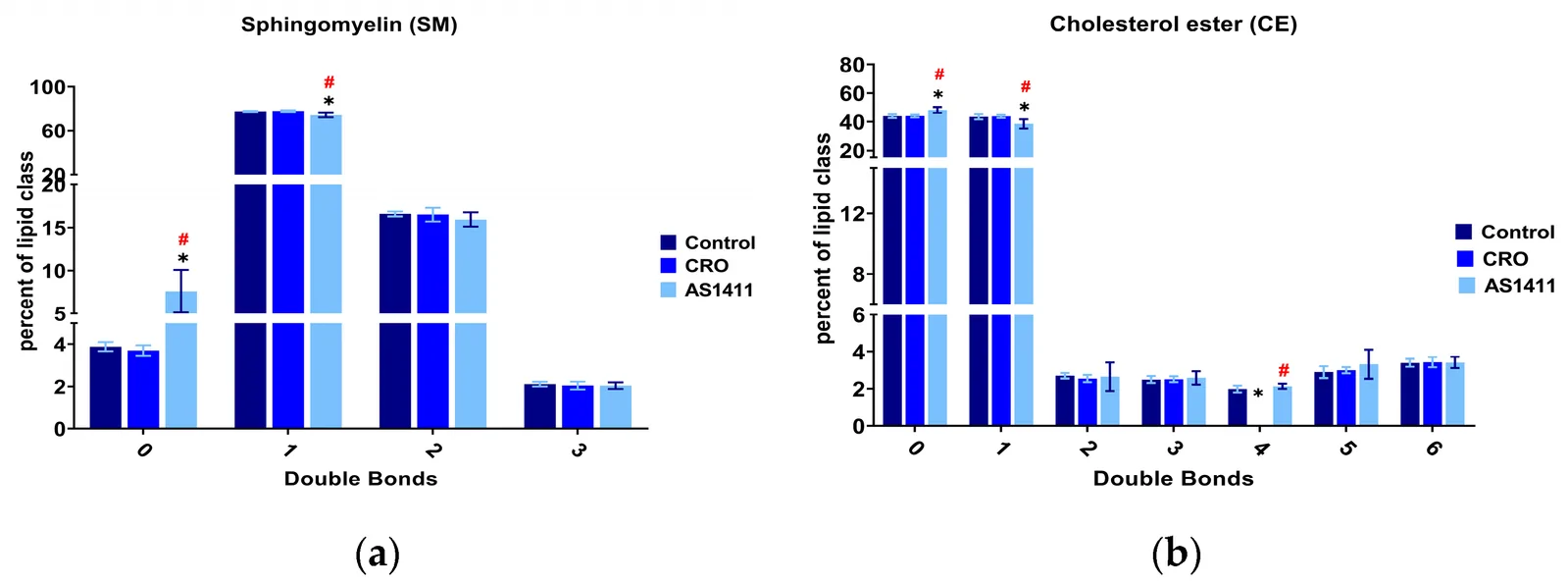
Total double bond profile of SP class including (**a**) SM and ST class including (**b**) CE in U-87 cells. * *p* < 0.05 vs. Control; # *p* < 0.05 vs. CRO.

The quantities of lipid species containing the same number of double bonds (DBs) were summed, normalized to the total amount of the respective lipid class and expressed as mol% of their respective classes (Figure 8, Figure 9 and Figure 10) (Tables S5 and S8). Following AS1411 treatment, a profound and sequence-specific biphasic remodeling of lipid saturation emerged across multiple classes (*p* < 0.05 vs. control and CRO). First, there was a systematic shift towards full saturation; while mono- and low-unsaturated species (DB = 1 to 4) were generally suppressed in the DAG (DB = 1, 2 and 4) (Figure 8a), TAG (DB = 1) (Figure 8b), PC (DB = 2) (Figure 9a), PE (DB = 1, 2, and 11) (Figure 9b), PI (DB = 3) (Figure 9d), SM (DB = 1) (Figure 10a) and CE (DB = 1) (Figure 10b) pools, the fully saturated (DB = 0) counterparts exhibited a significant compensatory increase, particularly in DAG (Figure 8a), TAG (Figure 8b), PG (Figure 9c), PI (Figure 9d), SM (Figure 10a) and CE (Figure 10b). Second, AS1411 uniquely triggered an extreme polyunsaturated species (PUFAs) accumulation. The highly PUFAs emerged exclusively in the TAG (DB = 6, 7) (Figure 8b), PC (DB = 7) (Figure 9a), and PG (DB = 8) (Figure 9c) after AS1411 treatment. The targeted PUFA enrichment was concurrently observed in the PE (DB = 4, 5, 6) (Figure 9b), PS (DB = 5) classes (Figure 9e). Statistical analysis revealed significant modulations in the levels of several distinct lipid species across multiple classes, specifically DAG (DB = 6) (Figure 8a), TAG (DB = 2 and 5) (Figure 8b), PI (DB = 2, 4, and 6) (Figure 9d), PS (DB = 1 and 4) (Figure 9e), and CE (DB = 4) (Figure 10b), following aptamer exposure. However, these alterations lacked AS1411 sequence specificity.

**Figure 11:**
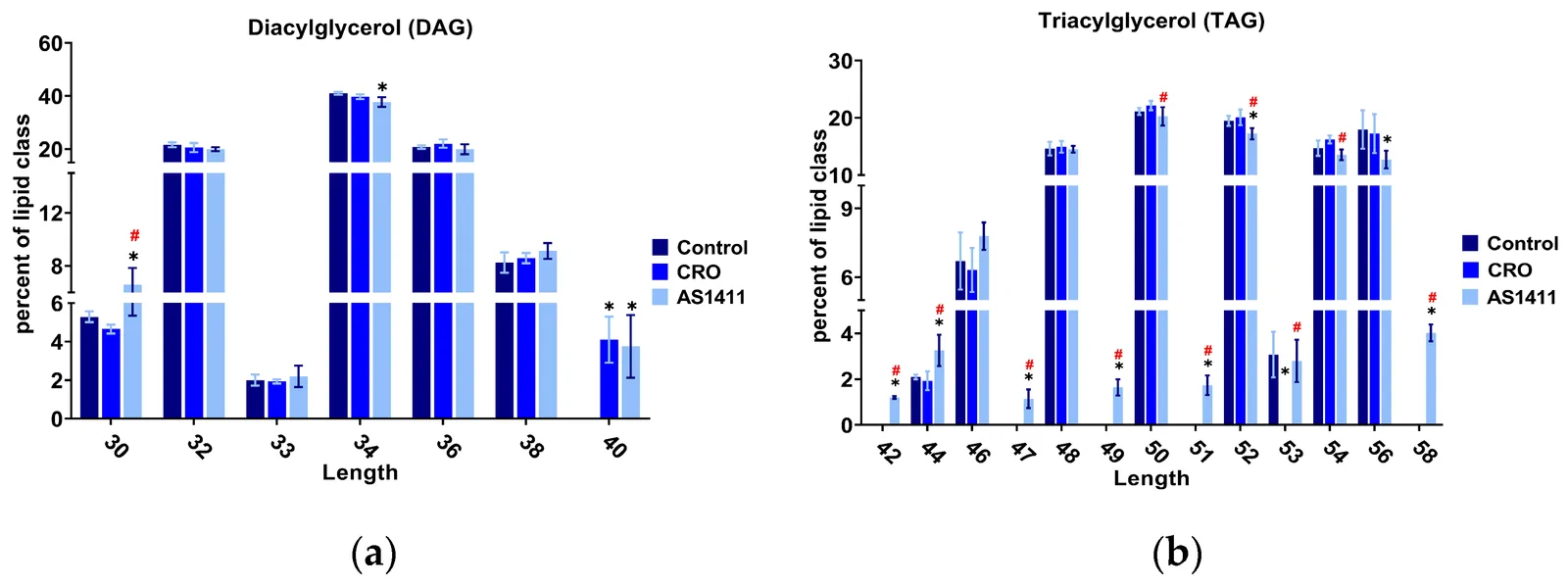
Total carbon chain length profile of GL class including (**a**) DAG and (**b**) TAG in U-87 cells. * *p* < 0.05 vs. Control; # *p* < 0.05 vs. CRO.

**Figure 12:**
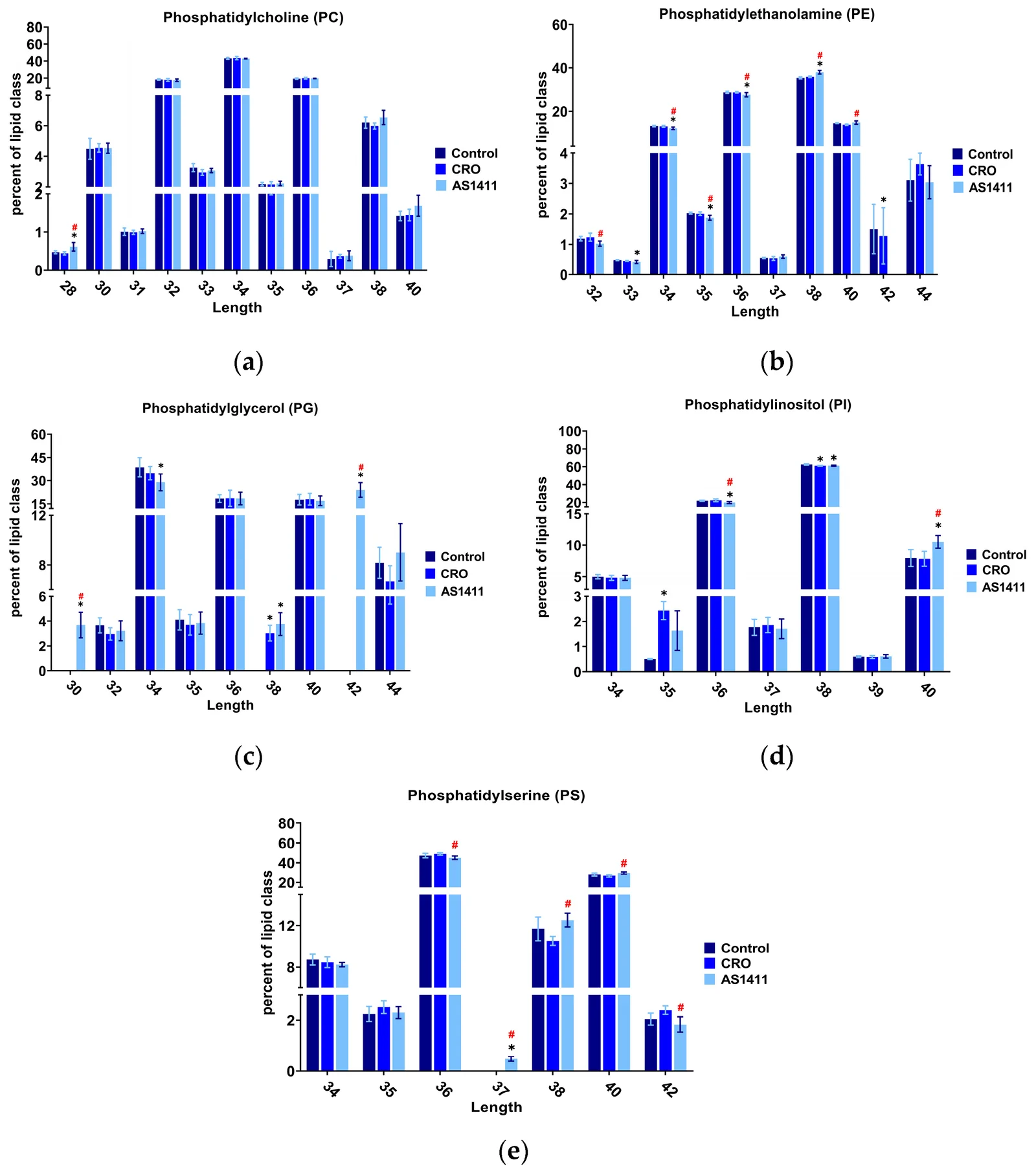
Total carbon chain length profile of GP class including (**a**) PC, (**b**) PE, (**c**) PG, (**d**) PI, and (**e**) PS in U-87 cells. * *p* < 0.05 vs. Control; # *p* < 0.05 vs. CRO.

**Figure 13:**
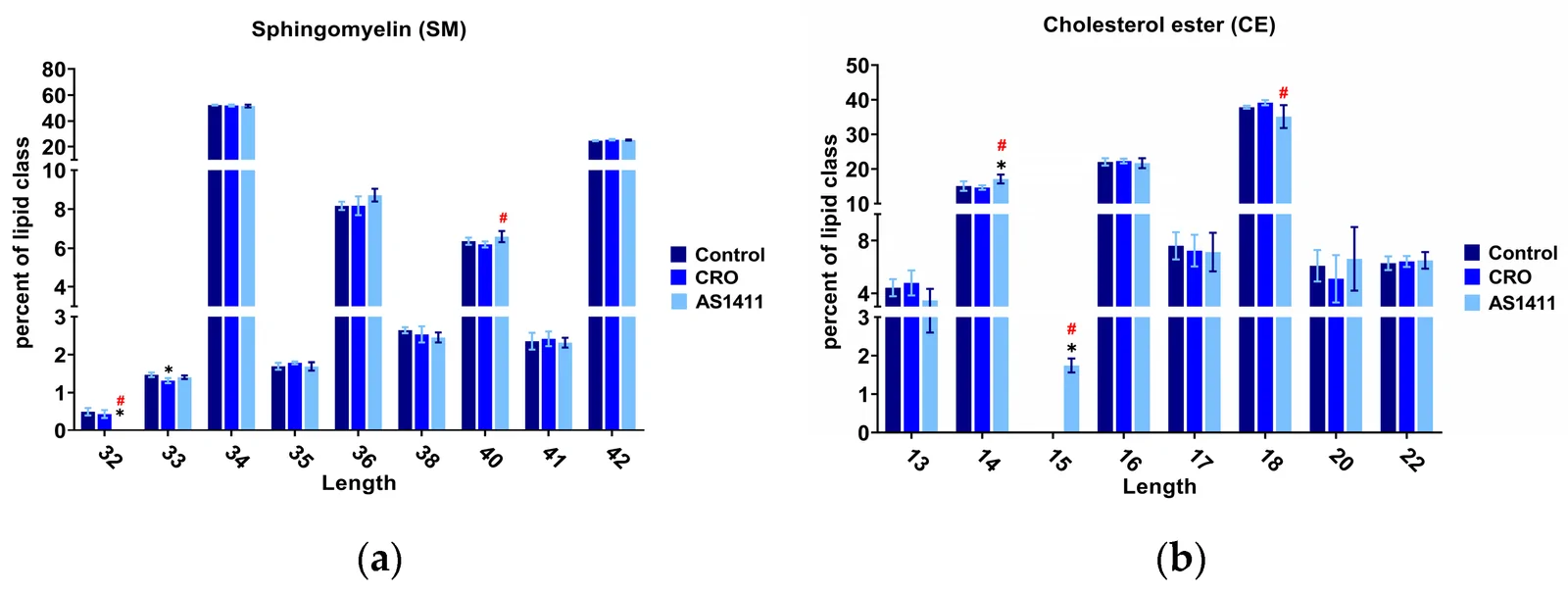
Total carbon chain length profile of SP class including (**a**) SM and ST class including (**b**) CE in U-87 cells. * *p* < 0.05 vs. Control; # *p* < 0.05 vs. CRO.

To evaluate acyl chain length remodeling, the quantities of lipid species containing the same number of carbon atoms in their hydrocarbon moiety were summed and normalized to the total amount of the given lipid class, expressed as mol% (Figure 11, Figure 12 and Figure 13) (Tables S5 and S9). Among DAG, the C30 exhibited a sequence-specific increase (Figure 11a). In the TAG pool, highly abundant C52 species were specifically depleted by AS1411, while TAG 44 demonstrated a significant increase (Figure 11b). Furthermore, the extremely long TAG 58, alongside longer species (TAG 42, 47, 49, and 51) that are normally absent, emerged exclusively after AS1411 treatment. Although no major chain length shift was observed in the PC pool, the sequence-specific accumulation of a very short species, PC 28, was notable (Figure 12a). Within the PE class, AS1411 specifically suppressed medium-chain species (C34–C36), whereas the long-chain PE 38 was significantly upregulated compared to both the control and CRO groups (Figure 12b). In PG pool, both the medium-chain PG 30 and the exceptionally long PG 42 appeared exclusively under AS1411 treatment (Figure 12c). For PI lipids, medium-chain species (PI 36) were specifically suppressed following AS1411 treatment, whereas long-chain counterparts (PI 40) demonstrated a significant sequence-specific increase (Figure 12d). The PS pool was characterized by the exclusive emergence of PS 37 (Figure 12e). Furthermore, SM 32 was decreased (Figure 13a), an odd-chain lipid, CE 15, emerged exclusively in the AS1411 group, accompanied by CE 14 accumulation (Figure 13b). DAG 34 and 40 (Figure 11a), TAG 50, 53, 54, and 56 (Figure 11b), PE 32, 33, 40, and 42 (Figure 12b), PG 34 and 38 (Figure 12c), PI 35 and 38 (Figure 12d), PS 36, 38, 40, and 42 (Figure 12e), SM 33 and 40 (Figure 13a), and CE 18 (Figure 13b) species were significantly altered following aptamer exposure. However, these alterations lacked AS1411 sequence specificity.

**Figure 14:**
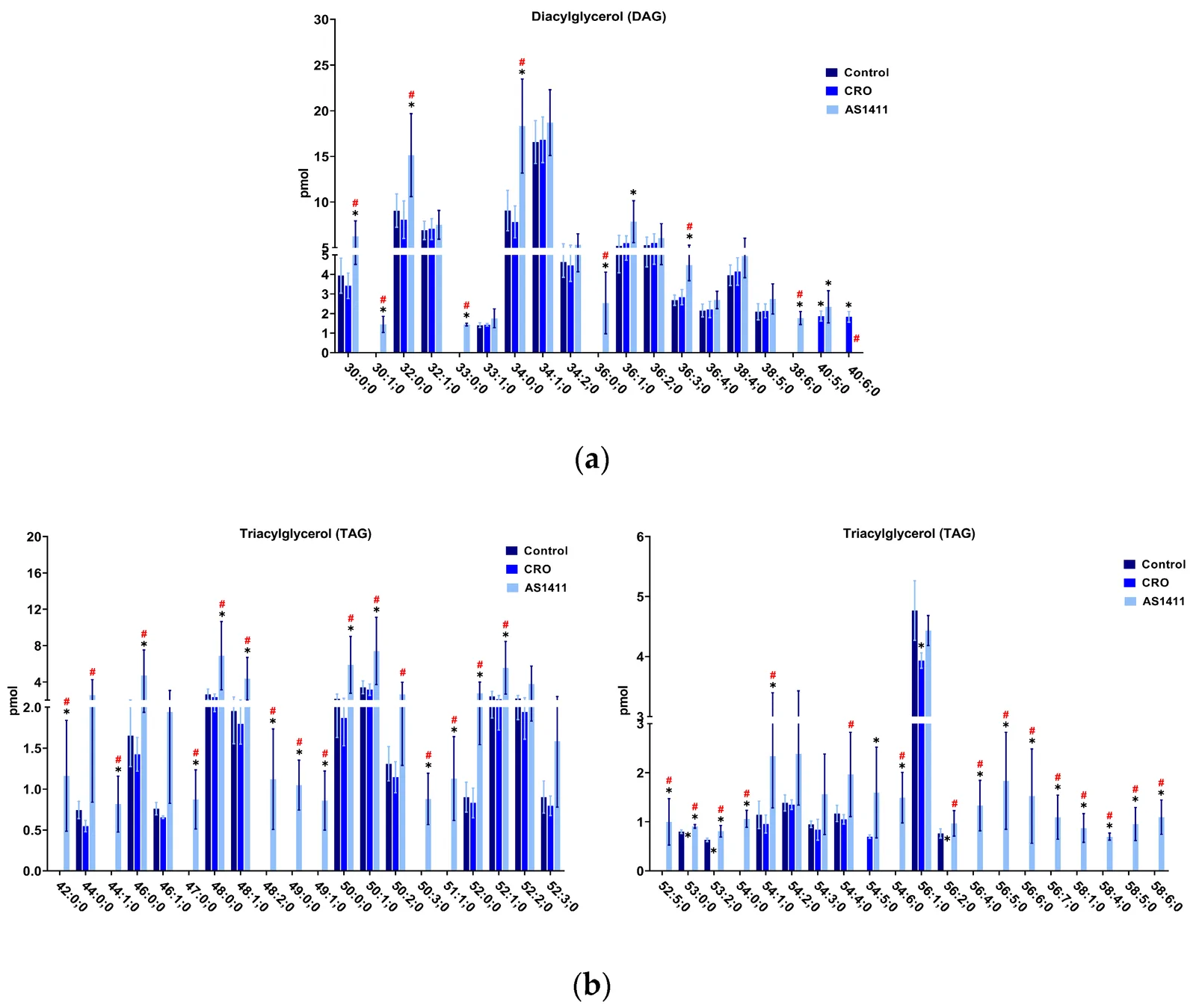
Lipid (sub-)species profile of GL class including (**a**) DAG and (**b**) TAG in U-87 cells. * *p* < 0.05 vs. Control; # *p* < 0.05 vs. CRO.

**Figure 15:**
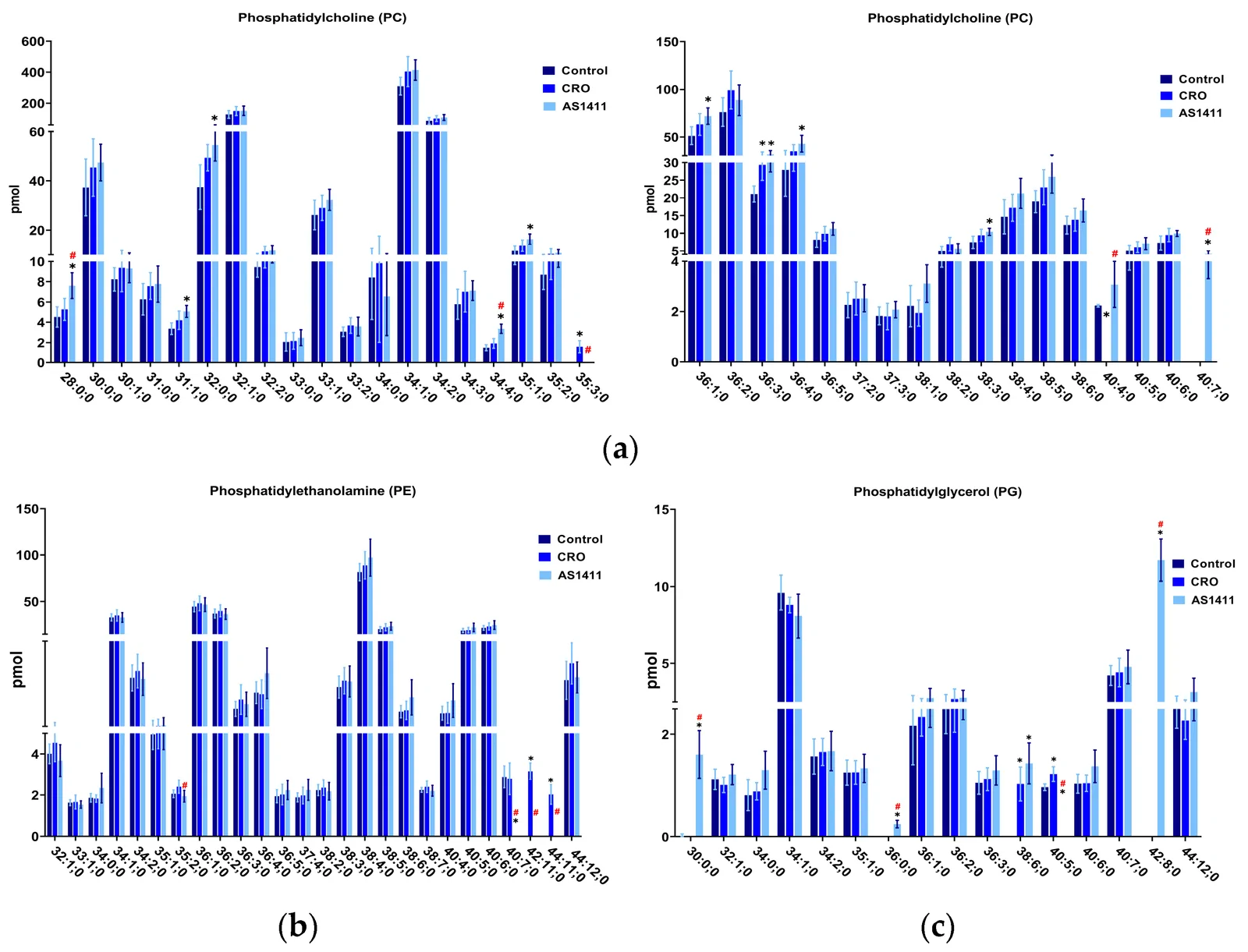

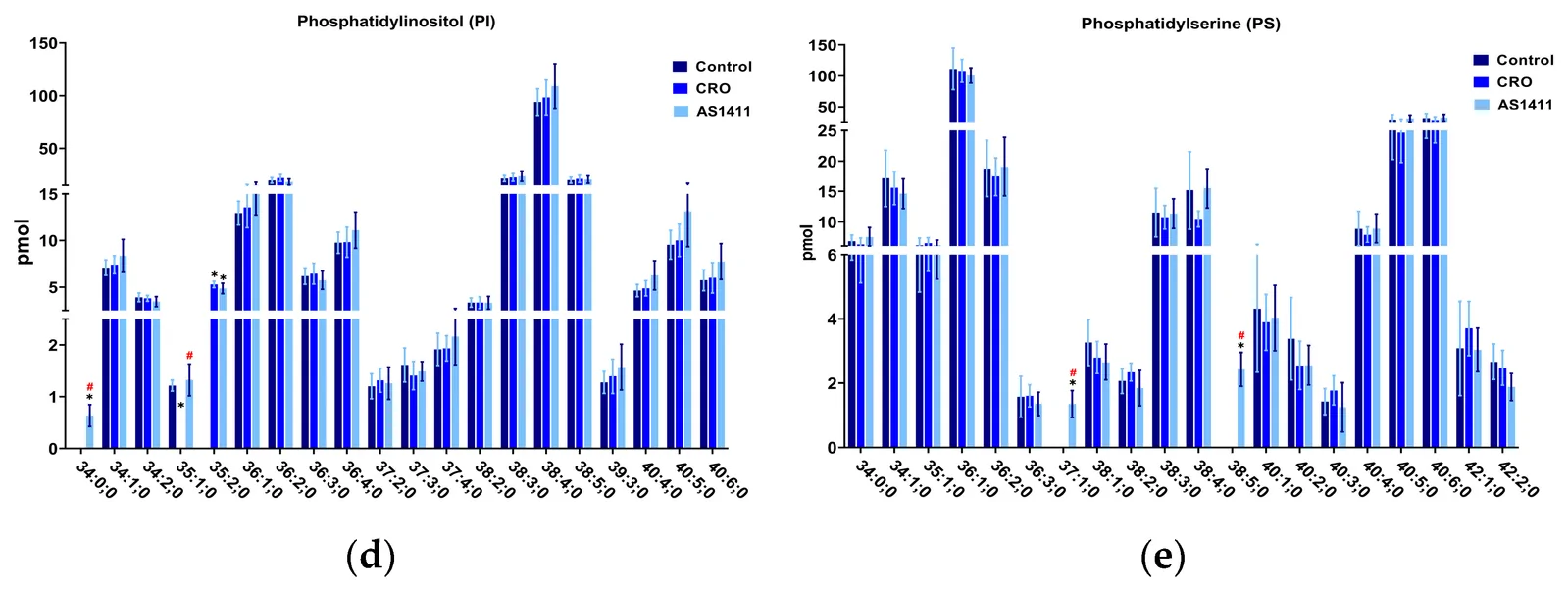
Lipid sub-species profile of GP class including (**a**) PC, (**b**) PE, (**c**) PG, (**d**) PI, and (**e**) PS in U-87 cells. * *p* < 0.05 vs. Control; # *p* < 0.05 vs. CRO.

**Figure 16:**
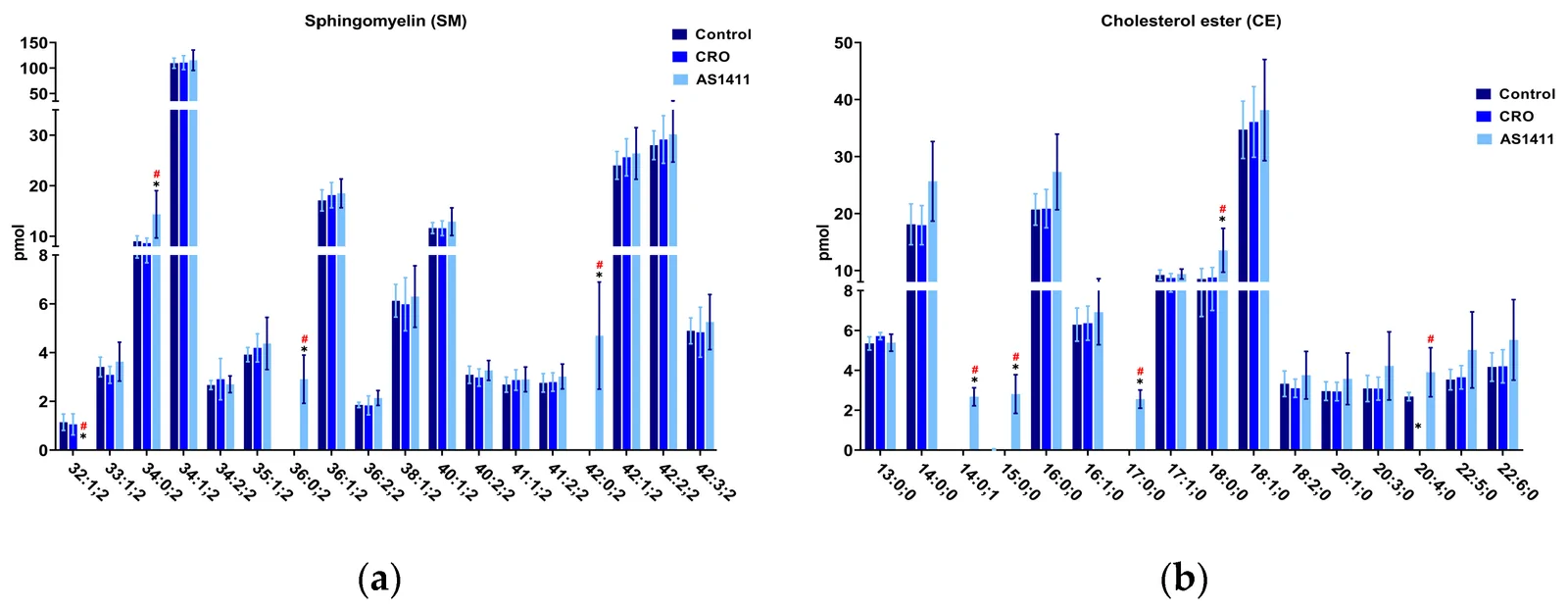
Lipid sub-species profile of SP class including (**a**) SM and ST class including (**b**) CE in U-87 cells. * *p* < 0.05 vs. Control; # *p* < 0.05 vs. CRO.

The lipid sub-species profile of analyzed samples are given as based on molar amount data (pmol) (Figure 14, Figure 15 and Figure 16) (Tables S6 and S10). Notably, certain species that are undetectable in the control group—specifically DAG 30:1;0, 33:0;0, 36:0;0, and 38:6;0—emerge exclusively following AS1411 treatment (Figure 14a). Additionally, several endogenous species (such as DAG 30:0;0, 32:0;0, 34:0;0, and 36:3;0) are significantly increased. Although DAG 36:1;0, 40:5;0, and 40:6;0 also exhibit alterations, these changes lack AS1411 specificity. The TAG 46:0;0, 48:0;0, 48:1;0, 50:0;0, 50:1;0, 52:0;0, 52:1;0, and 54:1;0 species are upregulated following AS1411 treatment. Furthermore, a distinct cluster of long-chain and highly polyunsaturated TAG species—which are virtually undetectable or present at very low levels in the control and CRO groups—is concurrently upregulated by AS1411 (Figure 14b). This cluster includes species such as 42:0;0, 44:1;0, 47:0;0, 48:2;0, 49:0;0, 49:1;0, 50:3;0, 51:1;0, 52:5;0, 54:0;0, 54:6;0, 56:4;0, 56:5;0, 56:6;0, 56:7;0, 58:1;0, 58:4;0, 58:5;0, and 58:6;0. The TAG 44:0;0, 50:2;0, 53:0;0, 53:2;0, 54:4;0, 54:5;0, 56:1;0, and 56:2;0 species exhibit alterations; nevertheless, all of these alterations lack AS1411 specificity (Figure 14b).

Within the PC class, AS1411 specifically induces the upregulation of PC 28:0;0 and 34:4;0, and the exclusive emergence of PC 40:7;0, whereas alterations observed in PC 31:1;0, 32:0;0, 35:1;0, 35:3;0, 36:1;0, 36:3;0, 36:4;0, 38:3;0, and 40:4;0 lack AS1411 specificity (Figure 15a). Notably, AS1411 induces a specific and significant decrease in PE 40:7;0, whereas the alterations observed in several other PE species (such as 35:2;0, 42:11;0, and 44:11;0) lack AS1411 specificity (Figure 15b). Within the PG class, certain species that are undetectable in the control group—specifically PG 30:0;0, 36:0;0, and 42:8;0—emerge exclusively following AS1411 treatment, whereas alterations observed in PG 38:6;0 and 40:5;0 lack AS1411 specificity (Figure 15c). While only PI 34:0;0 emerged exclusively following AS1411 treatment, the alterations observed in the 35:1;0 and 35:2;0 species lack AS1411 specificity (Figure 15d). In contrast, only the PS 37:1;0 and 38:5;0 species emerged exclusively following AS1411 treatment (Figure 15e).

Following AS1411 treatment, SM 32:1;2 is significantly downregulated whereas SM 34:0;2 is upregulated; furthermore, the SM 36:0;2 and 42:0;2 species emerged exclusively (Figure 16a). The CE pool demonstrates a sequence-specific modulation. Notably, certain esters—specifically CE 14:0;1, 15:0;0, and 17:0;0—emerged exclusively following AS1411 treatment. Concurrently, AS1411 induces a significant sequence-specific increase in CE 18:0;0. Finally, although CE 20:4;0 exhibited alterations, these changes lacked AS1411 sequence specificity (Figure 16b).

**Figure 17:**
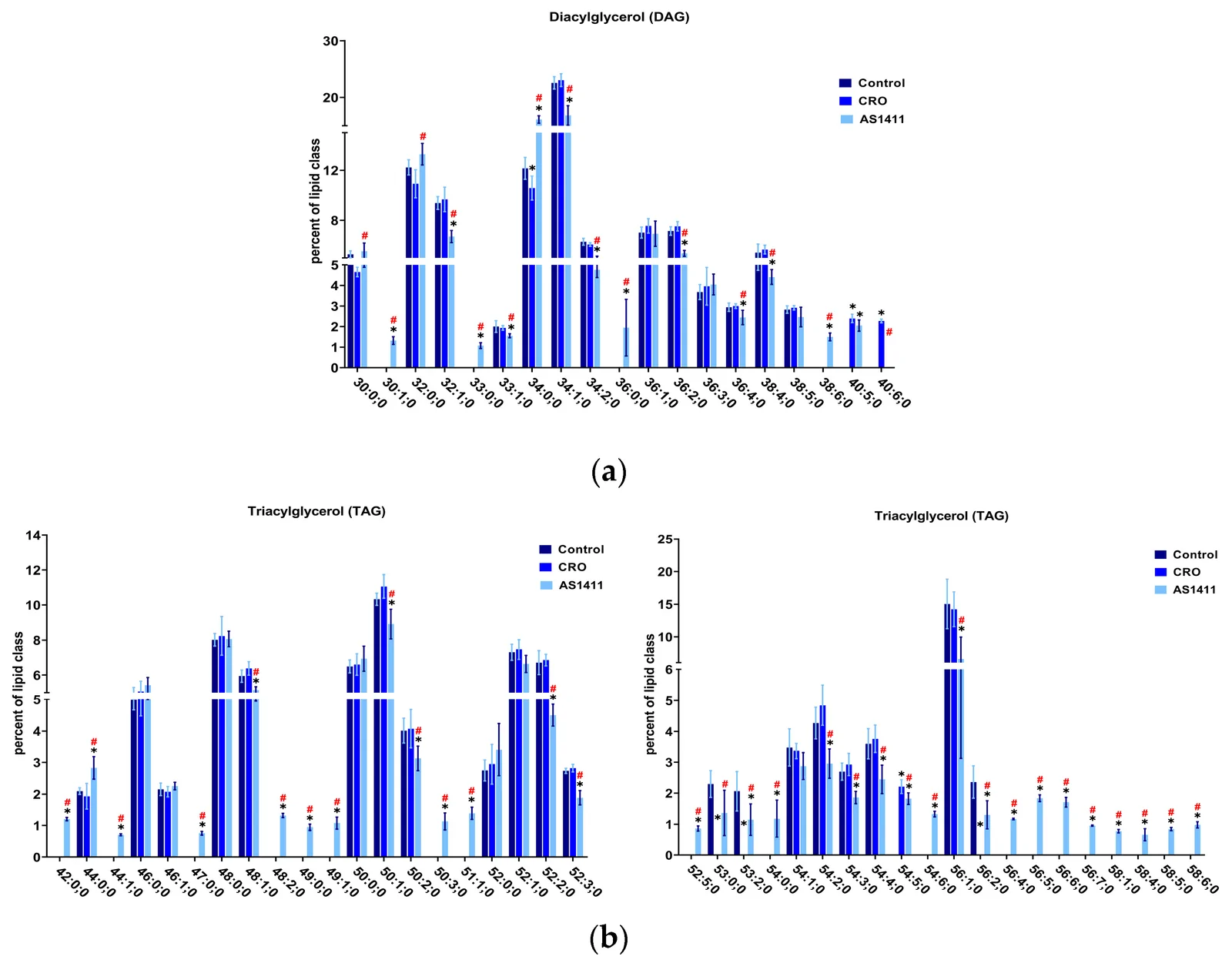
Lipid sub-species profile of GL class including (**a**) DAG and (**b**) TAG in U-87 cells. * *p* < 0.05 vs. Control; # *p* < 0.05 vs. CRO.

**Figure 18:**
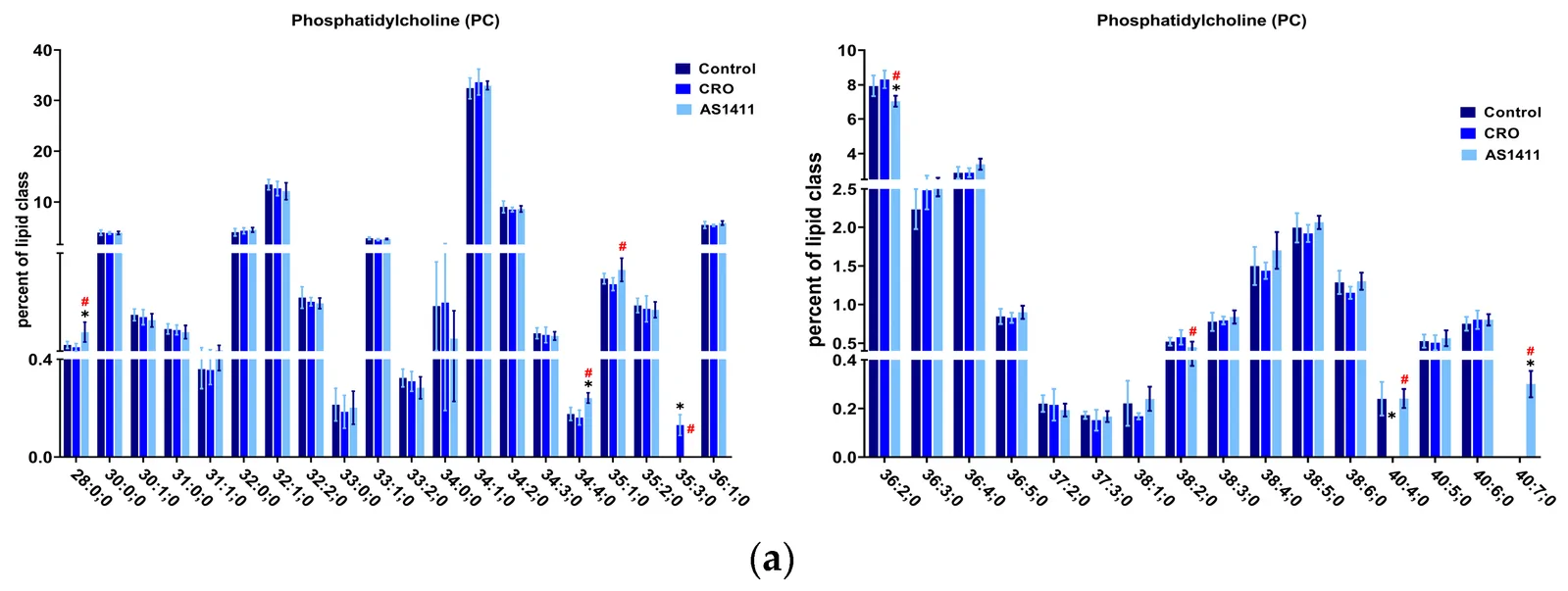

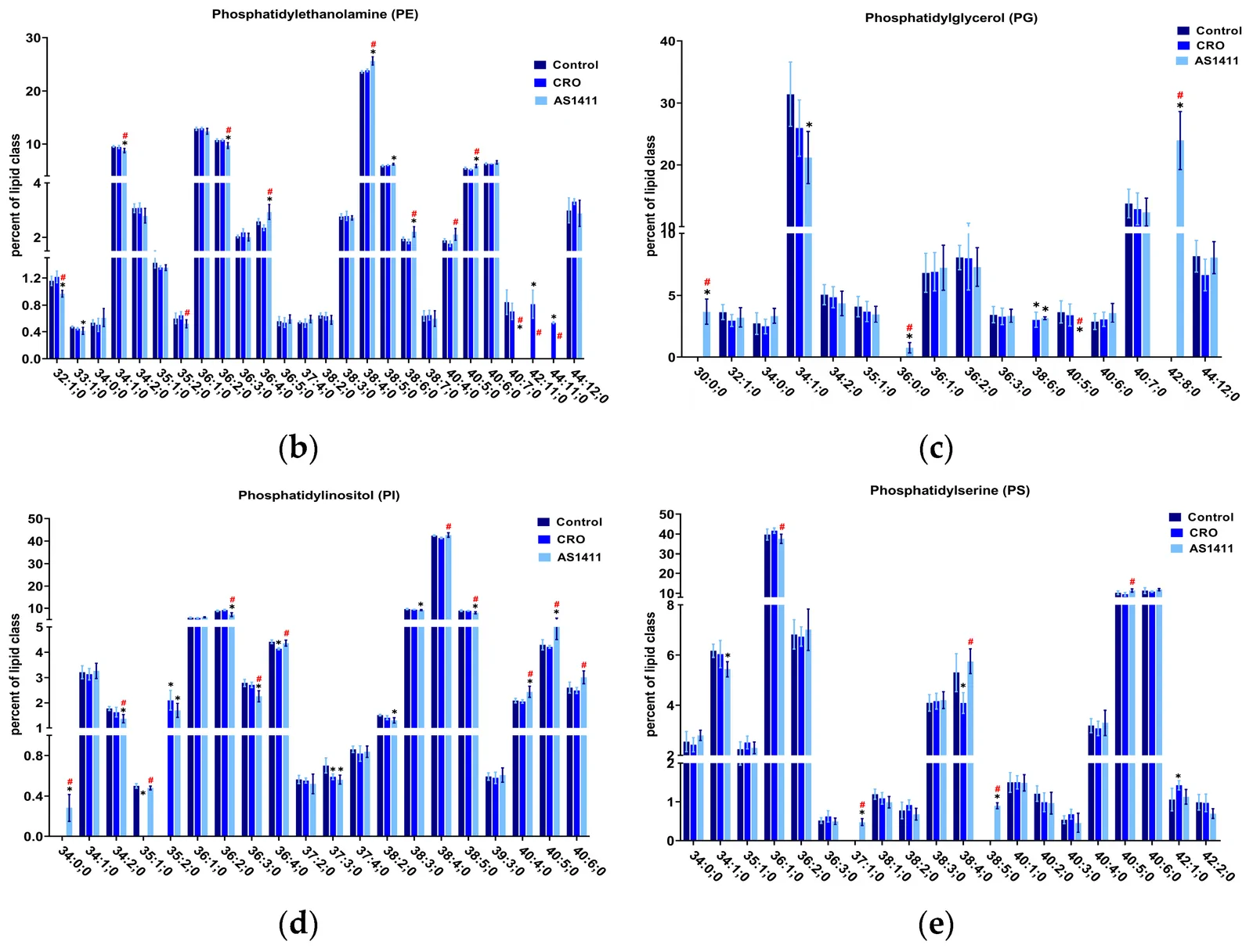
Lipid sub-species profile of GP class including (**a**) PC, (**b**) PE, (**c**) PG, (**d**) PI, and (**e**) PS in U-87 cells. * *p* < 0.05 vs. Control; # *p* < 0.05 vs. CRO.

**Figure 19:**
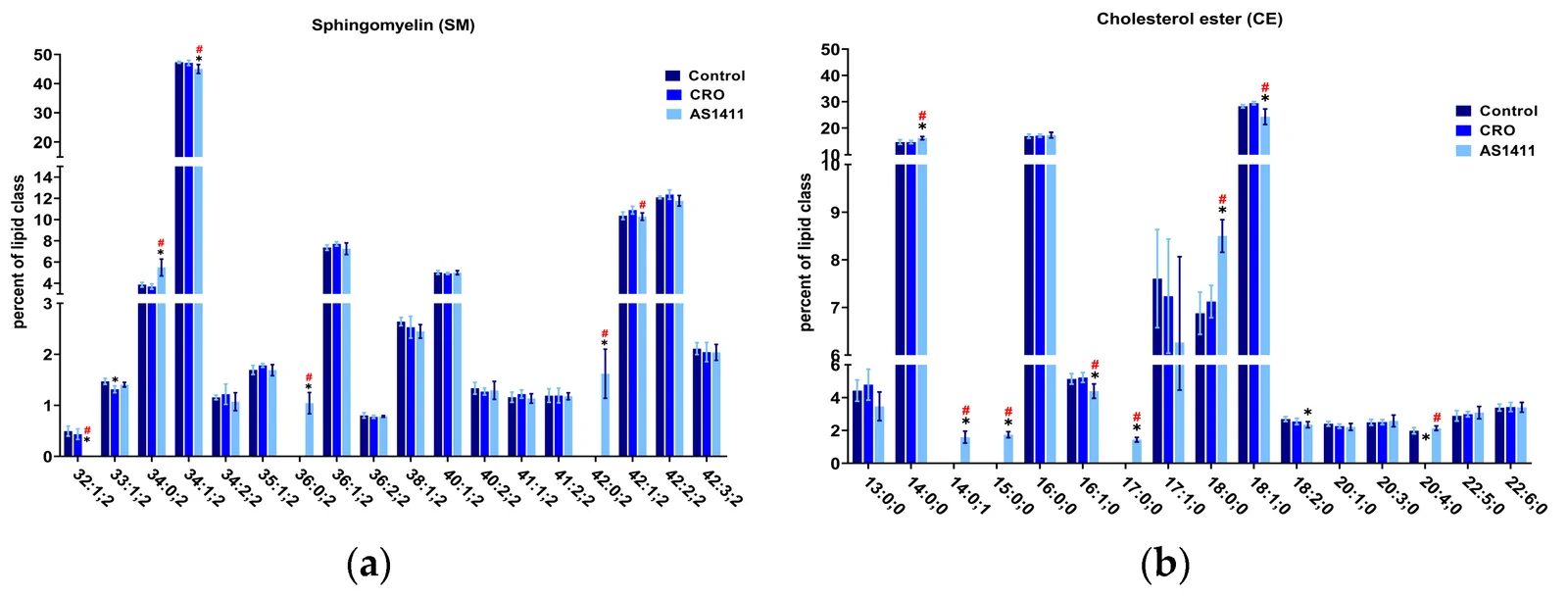
Lipid sub-species profile of SP class including (**a**) SM and ST class including (**b**) CE in U-87 cells. * *p* < 0.05 vs. Control; # *p* < 0.05 vs. CRO.

The lipid sub-species profile of analyzed samples are given based on data standardized to the lipid class (mol%) (Figure 17, Figure 18 and Figure 19) (Tables S7 and S11). Notably, certain species that are undetectable in the control group—specifically DAG 30:1;0, 33:0;0, 36:0;0, and 38:6;0—emerge exclusively following AS1411 treatment. Concurrently, several endogenous species (such as DAG 32:1;0, 33:1;0, 34:1;0, 34:2;0, 36:2;0, 36:4;0, and 38:4;0) are significantly suppressed. In contrast, although DAG 30:0;0, 32:0;0, 34:0;0, 40:5;0, and 40:6;0 also exhibit alterations, these changes lack AS1411 specificity (Figure 17a). The TAG 44:0;0 species and also, a distinct cluster of long-chain and highly polyunsaturated TAG species—which are virtually undetectable or present at very low levels in the control and CRO groups—is upregulated by AS1411 (Figure 17b). This cluster includes species such as 42:0;0, 44:1;0, 47:0;0, 48:2;0, 49:0;0, 49:1;0, 50:3;0, 51:1;0, 52:5;0, 54:0;0, 54:6;0, 56:4;0, 56:5;0, 56:6;0, 56:7;0, 58:1;0, 58:4;0, 58:5;0, and 58:6;0. Simultaneously, AS1411 specifically downregulated several previously abundant TAG species (e.g., TAG 48:1;0, 50:1;0, 50:2;0, 52:2;0, 52:3;0, 54:2;0, 54:3;0, 54:4;0, and 56:1;0) in a sequence-specific manner (Figure 17b). Although the levels of TAG 53:0;0, 53:2;0, 54:5;0, and 56:2;0 are altered, these changes lack AS1411 specificity. Within the PC class, AS1411 specifically induces the upregulation of PC 28:0;0 and 34:4;0, the depletion of PC 36:2;0, and the exclusive emergence of PC 40:7;0, whereas alterations observed in PC 35:1;0, 35:3;0, 38:2;0, and 40:4;0 lack AS1411 specificity (Figure 18a). Similar to PC, the PE pool exhibits an adaptive chain-elongation signature. Shorter and unsaturated PE species (e.g., 32:1;0, 34:1;0, 36:2;0, and 40:7;0) are significantly downregulated, whereas longer and highly unsaturated species (e.g., 36:4;0, 38:4;0, 38:6;0, and 40:5;0) are specifically elevated by AS1411. Furthermore, the alterations observed in PE 33:1;0, 35:2;0, 38:5;0, 40:4;0, 42:11;0, and 44:11;0 lack AS1411 specificity (Figure 18b). Within the PG class, certain species that are undetectable in the control group—specifically PG 30:0;0, 36:0;0, and 42:8;0—emerge exclusively following AS1411 treatment, whereas alterations observed in PG 34:1;0 and 38:6;0 lack AS1411 specificity.

Notably, only the PG 40:5;0 species demonstrates an AS1411-specific decrease (Figure 18c). PI species (such as 34:2;0, 36:2;0, 36:3;0, and 38:5;0) are specifically downregulated by AS1411. In stark contrast, long-chain polyunsaturated PI species (e.g., 40:4;0 and 40:5;0) are significantly enriched in the AS1411-treated group, accompanied by the exclusive emergence of the specific shorter species PI 34:0;0. Conversely, although the PI 35:1;0, 35:2;0, 36:4;0, 37:3;0, 38:2;0, 38:3;0, 38:4;0, and 40:6;0 species exhibit alterations, these changes lack AS1411 specificity (Figure 18d). Within the PS pool, although several species (e.g., PS 34:1;0, 36:1;0, 38:4;0, 40:5;0, and 42:1;0) exhibit alterations, these changes lack AS1411 specificity. In contrast, only the PS 37:1;0 and 38:5;0 species emerged exclusively following AS1411 treatment (Figure 18e). While the major unsaturated SM species that comprise the bulk of the pool remain largely stable, with the notable exception of a decrease in SM 32:1;2 and 34:1;2, AS1411 induces a specific and significant upregulation of the fully saturated SM 34:0;2 species. Furthermore, the SM 36:0;2 and 42:0;2 species, which are undetectable in the control group, emerged exclusively following AS1411 treatment. Conversely, alterations observed in the SM 33:1;2 and 42:1;2 species lack AS1411 specificity (Figure 19a). The CE pool demonstrates a sequence-specific modulation. Notably, certain esters—specifically CE 14:0;1, 15:0;0, and 17:0;0—emerged exclusively following AS1411 treatment. Concurrently, AS1411 induces a significant sequence-specific increase in other species (e.g., CE 14:0;0 and 18:0;0), while driving a significant decrease in specific esters (e.g., CE 16:1;0 and 18:1;0). Finally, although CE 18:2;0 and 20:4;0 exhibited alterations, these changes lacked AS1411 sequence specificity (Figure 19b).

## 4. Discussion

Nucleolin (NCL) is a multifunctional RNA-binding protein that plays a key role in several cellular processes within eukaryotic cells, including cell division, survival, ribosome biogenesis, and cell size regulation. Under normal physiological conditions, this protein is predominantly localized within the nucleolus; however, it is upregulated in cancer cells undergoing oncogenic transformation and translocates to the cytoplasm and the cell surface (plasma membrane) [56]. This altered localization on the plasma membrane renders NCL an ideal and highly specific molecular receptor for targeted anticancer therapies [57]. AS1411 is a guanosine-rich, 26-nucleotide G-quadruplex DNA aptamer that specifically binds to the glycine-arginine-rich (GAR) domain at the C-terminus of NCL [56].

Our cell survival assays at 24 h demonstrate a clear, sequence-specific, and concentration-dependent reduction in U-87 glioblastoma cell survival following AS1411 treatment. This acute activity profile suggests that AS1411 rapidly engages its molecular targets, with the time-dependent decline potentially reflecting cellular adaptation mechanisms or aptamer degradation. Crucially, the CRO exerted minimal effects on cell viability across the entire concentration gradient (1–80 µM) (Figure 1). This stark divergence confirms that the observed cytotoxicity is not a generic artifact of polyanionic macromolecule internalization or carrier toxicity, but a highly targeted, NCL-dependent pharmacological event.

The lack of a statistically massive accumulation in total lipid mass (Figure 2) proves that AS1411 is not a general toxin or a random lipogenic agent that uncontrollably overloads the cell with lipids. It confirms that the dramatic changes detected in the species-level analyses (e.g., the exclusive emergence of specific long-chain TAGs or polyunsaturated GPs (PG, PG, Ps, and PI) occur without inflating the total bulk lipid mass of the cell, proceeding strictly through targeted remodeling and an adaptive scaffolding response.

Principal Component Analysis (PCA) is an unsupervised statistical method that enables the dimensionality reduction, visualization, and interpretation of complex, multidimensional large datasets (e.g., lipidomics, transcriptomics). PCA mathematically computes the directions containing the greatest variance (variability) in the dataset and designates these as Principal Components (PCs). The highest variance is represented on the PC1 axis, while the second-highest variance is represented on the PC2 axis. In lipidomic analyses, PCA provides the holistic metabolic response of cells with a treatment. The scores plot maps (Figure 3a and Figure 4a) the overall cellular impact of the treatment and biological variations by showing how experimental groups (e.g., Control, CRO, and AS1411) cluster together or separate from each other. The loadings plot (Figure 3b and Figure 4b) determines which specific lipid species (variables) drive this separation (or clustering). In the PCA model based on absolute lipid quantities (Figure 3), the control and CRO groups exhibit relatively close and homogeneous clustering in the center–left region of the plot. In contrast, the group treated with AS1411 (Sample/Apt) demonstrates a distinct shift toward the positive (right) side along the PC1 axis, albeit with a high intra-group distribution (biological variance) (Figure 3a). When examining the loadings plot (Figure 3b), it is clearly evident that the main driving force pulling the AS1411 group toward the positive (right) direction of PC1 is the TAG species, which form a dense cluster in the lower right corner of the plot. Additionally, certain DAG and CE species significantly contribute to this separation. This indicates that the absolute biomass increase and high inter-cellular variance induced by AS1411 primarily stem from the massive, exclusively emerged neutral lipids (TAG/DAG), indicating that the cellular response revolves around a lipotoxic accumulation. PCA findings based on relative distribution (mol%) data reflect the specific remodeling within the internal structural balance of cellular membranes and lipid pools, independent of absolute mass increase (volumetric expansion) (Figure 4). While the control and CRO groups tightly cluster together on the positive (right) side of the PC1 axis, the AS1411 (Sample/Apt) group is completely and distinctly shifted to the negative (left) side of the PC1 axis. This absolute left–right segregation demonstrates that the cellular effect generated by AS1411 is not a random lipid accumulation (Figure 4a). The loadings plot (Figure 4b) illustrates that the lipid species pulling the AS1411 group (toward the negative/left side of PC1) trace a highly specific profile. This region is populated by long-chain/polyunsaturated PE and specific PG species representing structural adaptation, alongside particular TAG and DAG subclasses representing neutral lipid remodeling. Conversely, the positive (right) side of PC1 is heavily weighted by standard structural lipids (mostly PC and PI species) that maintain the basal state of control cells. This architecture suggests that AS1411 induces specific elongase/desaturase modifications in the cell membrane, establishing a targeted, sequence-dependent cellular stress scaffolding.

In high-throughput “omic” analyses, the Volcano Plot serves as a fundamental analytical tool to identify and visualize the most critical biological signals within massive datasets. The significance of this analysis lies in its ability to simultaneously map two essential statistical criteria across thousands of molecules (lipid species in this study): the biological effect size (Fold Change on the X-axis) and the statistical reliability (−log10(*p*-value) on the Y-axis). This dual visualization enables the unbiased and rapid filtering of molecules that are not only statistically significant but also exhibit a magnitude of change substantial enough to alter the cellular phenotype. In a plot that maps the global impact of the AS1411 treatment on the cellular lipidome (Figure 5), two distinct Analyzing the Volcano metabolic populations emerge. The green data points represent specific lipid species that are significantly upregulated (Fold Change > 1), indicating substantial accumulation following treatment. The high density of green points reaching values of 4.0 and above on the Y-axis confirms that the cellular system is not experiencing random alterations; rather, the treatment exerts high statistical power, triggering a targeted and massive lipid synthesis storm. Conversely, the blue data points denote the downregulated lipid species (Fold Change < 1), representing the lipid pools that are suppressed by the aptamer.

### 4.1. Lipid Categories, Lipid Classes, Double Bond (DB) Number, and Carbon Chain Length

A critical situation emerges when transitioning from macro-level classes to the individual lipid species level: specific lipid species exhibit profound, sequence-specific significance under AS1411 treatment, even within lipid categories that appear non-significant or CRO-driven at the macro level. This discrepancy is not a contradiction; rather, it highlights intense intra-class remodeling. When AS1411 induces the downregulation of certain endogenous species (e.g., short-chain/saturated species) while simultaneously forcing the exclusive emergence or upregulation of others (e.g., extreme PUFA species); these bidirectional alterations effectively cancel each other out in the total class sum (Tables S10 and S11). Consequently, the bulk pool size remains statistically stable or mirrors generic stress, masking the significant acyl-chain restructuring occurring underneath. Therefore, analyzing the lipidome solely at the macro level obscures the true pharmacological impact; the targeted nature of AS1411 only becomes fully visible at the micro-species level, where sequence-specific saturation and chain-length alterations are actively driving membrane destabilization and lipotoxicity.

At the macro-level of lipid categories, AS1411 induced a sequence-specific redirection of intracellular lipid fluxes. The most striking quantitative alteration is the accumulation of GL, which is significantly elevated exclusively in the AS1411 group compared to both the control (* *p* < 0.05) and the CRO group (# *p* < 0.05) (Figure 6a,b). Conversely, while GP—the primary building blocks of the cellular membrane—demonstrates a sequence-specific depletion, ST exhibits a significant relative increase against the CRO group (# *p* < 0.05) (Figure 6b). Resolving these broad categories into specific lipid classes allowed for a more detailed evaluation of the lipid alterations (Figure 7a,b). The GL accumulation is fundamentally driven by a dual enrichment of DAG and TAG. Both of these neutral lipid classes are significantly upregulated by AS1411 relative to both the control (* *p* < 0.05) and CRO (# *p* < 0.05) groups (Figure 7a,b). Within the structural phospholipid compartment, PE and PS exhibit a significant depletion specifically under AS1411 treatment compared to the control (* *p* < 0.05), while PA is uniquely elevated in the AS1411 cohort (Figure 7b). The significant class-level alterations observed in PC are primarily driven by the CRO group (* *p* < 0.05 vs. control) whereas, CE exhibits a significant relative increase against the CRO group (# *p* < 0.05) (Figure 7b).

Evaluating aptamer-induced lipidomic changes requires rigorously separating sequence-specific pharmacological effects from the generalized biophysical stress of nucleic acid delivery. Our analysis revealed that a subset of double bonds, acyl chain length and lipid sub-species alterations lacks AS1411 specificity, instead reflecting a common cellular adaptation to foreign oligonucleotide uptake (Figure 6, Figure 7, Figure 8, Figure 9, Figure 10, Figure 11, Figure 12, Figure 13, Figure 14, Figure 15, Figure 16, Figure 17, Figure 18 and Figure 19) (Tables S8 and S11). Recognizing and filtering out these generic biophysical stress responses—rooted in the mechanical and metabolic toll of endosomal aptamer processing—is crucial. By eliminating this background noise, the true sequence-specific mechanistic impact of AS1411 becomes evident, setting the stage for evaluating the profound structural disruptions in acyl chain dynamics, particularly regarding chain length remodeling.

Despite the therapeutic potential of AS1411, the molecular mechanisms by which it regulates cell size and metabolism—particularly its effects on the biosynthesis and homeostasis of lipids, the building blocks of cellular membranes—have remained unclear until recently. Lipids, the fundamental building blocks of cell membranes, are not merely passive structural barriers enclosing organelles but are dynamic bioactive molecules that directly coordinate membrane protein activity, intracellular signaling cascades, energy storage, and cell death mechanisms [58]. Analysis of the double bond (DB) and carbon chain length datasets obtained from CRO and AS1411-treated cells clearly demonstrates that the cancer cell lipidome does not undergo random deformation; rather, it enters a targeted biochemical remodeling process that develops in a sequence-specific manner as a response to pharmacological stress.

#### 4.1.1. DAG Dynamics and Signal Transduction

The number of double bonds in the acyl chains of lipids is a critical physicochemical parameter that directly determines membrane fluidity, sensitivity to oxidative stress, and targeted protein–lipid interactions [59]. Data obtained for the DAG class show an increase in DB = 0 species, a decrease in DB = 1, 2, and 4 species (Figure 8a). Regarding carbon chain lengths, C30 increases, while non-sequence-specific changes are detected in C34 and C40 species (Figure 11a). DAG is a potent second messenger located in the lipid bilayer of cellular membranes that can alter intracellular signaling cascades within seconds, particularly by activating the Protein Kinase C (PKC) family [60]. The increase in fully saturated (DB = 0) and relatively short-chain (C30) DAG species upon AS1411 application suggests that the cell structurally remodels its DAG pool to facilitate membrane curvature during endosomal trafficking or macropinocytosis [61].

#### 4.1.2. Neutral Lipid Storage and Lipotoxicity Buffering in the TAG Class

TAG are the main components of neutral lipid droplets where cellular energy is stored, and they function as buffers against cellular lipotoxicity [62]. In the TAG dataset, an increase in DB = 0 (fully saturated) species, a decrease in DB = 1 species, while species bearing a high degree of polyunsaturation (DB = 6 and 7) appear sequence-specifically in the AS1411-treated groups (Figure 8b). The chain length profile supports this trend; C52 species downregulated, whereas C44 increases, and specific molecules such as C42, C47, C49, C51, and C58 (some containing an odd number of carbons) appear following AS1411 treatment (Figure 11b). Under severe cellular stress, particularly when threatened by reactive oxygen species accumulation and oxidative stress, cancer cells avoid maintaining reactive PUFAs in a free state or within structural phospholipid forms in cell membranes [59]. It is established that AS1411 induces methuosis, a non-apoptotic cell death pathway characterized by macropinocytosis hyperstimulation in target cells [63]. During methuosis, the cell requires substantial amounts of new membrane to form large vacuoles; however, these vacuoles fail to fuse with lysosomes and accumulate in the cytoplasm [64]. The reactive PUFAs (DB = 6, 7) released or synthesized during this chaotic cycle of membrane synthesis and degradation are rapidly esterified into TAG molecules and directed toward the inert core of cytoplasmic lipid droplets to protect the plasma membrane and organelles from lipid peroxidation [65]. The appearance of odd-chain lengths, such as C47, C49, and C51, indicates that AS1411-induced metabolic stress alters the specificity of lipogenic enzymes, possibly by activating peroxisomal alpha-oxidation pathways or alternative substrate utilization like propionyl-CoA.

#### 4.1.3. Biochemical Basis of PE and Ferroptosis Sensitivity

Analysis of experimental saturation data for the PE class shows that saturated or monounsaturated species (DB = 1, 2, 11) are sequence-specifically downregulated, whereas highly polyunsaturated PE species (DB = 4, 5, 6) increase and become enriched (Figure 9b). The carbon chain length data support this finding, indicating that relatively short-chain PE species (C34, C35, C36) are downregulated, while longer-chain species (C38) increase (Figure 12b). Literature data confirm that AS1411 application leads to an approximate two fold increase in total PE levels in NIH-3T3 cells [56]. Our results demonstrate that this mass increase is not random but specifically targets PEs containing long-chain polyunsaturated fatty acids (PUFAs) (C38 and DB = 4, 5, 6). In a biological and pathological context, PEs enriched with long-chain PUFAs, such as arachidonic acid (AA, C20:4) and adrenic acid (AdA, C22:4), serve as primary substrates for ferroptosis, an iron-dependent and lethal lipid peroxidation process [66]. Under normal conditions, these PUFAs are activated by Acyl-CoA Synthetase Long-Chain Family Member 4 (ACSL4) and integrated into the PE backbone by the LPCAT3 enzyme; they are subsequently oxidized by lipoxygenases (particularly ALOX15), leading to catastrophic plasma membrane disruption [66]. The ability of AS1411 to sensitize cells to ferroptosis—and to trigger synergistic ferroptotic death *in vivo* and *in vitro* when conjugated with agents like erianin (AS1411-BSA@ERN nanocarriers)—is documented in the literature [67]. The increase in PE DB = 4, 5, 6 and C38 in the experimental data provides clear evidence at the cellular level that the AS1411 aptamer pharmacologically primes the cancer cell for ferroptosis.

#### 4.1.4. Dynamics of PC, PG, PI, and PS

The saturation and length profiles of matrix phospholipids are directly related to macropinocytosis and organelle integrity. In the PC class, short-chain C28 increases, and a highly unsaturated species (DB = 7) appears sequence-specifically in the AS1411-treated groups (Figure 9a and Figure 12a). In the PG class, DB = 0 increases, and species with a DB = 8 degree of unsaturation and C30 or C42 carbon lengths emerge following AS1411 treatment (Figure 9c and Figure 12c). PG is synthesized predominantly in the inner leaflet of mitochondrial membranes rather than cellular membranes and serves as a precursor for cardiolipin biosynthesis [68]. The appearance of larger, unsaturated molecules like PG DB = 8 and C42 in mitochondrial membranes suggests that AS1411 induces structural stress on the mitochondrial electron transfer chain or membrane potential [56]. In the PI class—a regulator of endosomal trafficking and lysosomal fusion processes—C36 decreases while C40 increases; regarding saturation, DB = 0 increases while DB = 3 decreases (Figure 9d and Figure 12d). PI metabolism, particularly phosphoinositide signaling operating through the PIKFYVE lipid kinase, is required for the shrinkage, lysosomal trafficking, and acidification of macropinosomes [61]. Given that AS1411 exposure triggers macropinocytosis and results in vacuole accumulation (methuosis) in cancer cells, shifting the PI pool toward long-chain (C40) and saturated forms may act as a structural blockage mechanism that disrupts vesicular trafficking and impedes macropinosome–lysosome fusion [63]. Finally, in the PS class, which maintains plasma membrane asymmetry and is normally restricted to the inner leaflet, an increase in DB = 5 and the appearance of C37 length species are observed (Figure 9e and Figure 12e). The translocation of PS to the outer leaflet typically serves as an apoptotic marker that triggers phagocytosis by macrophages [68]. This specific PUFA enrichment in PS profiles suggests that AS1411 disrupts membrane asymmetry, potentially altering immune evasion mechanisms or tumor microenvironment signaling in the cancer cell.

#### 4.1.5. CE Synthesis, the Cholesterol Paradox, and SM-Mediated Membrane Fluidity

Experimental analysis of the CE class indicates that fully saturated (DB = 0) species increase and DB = 1 species decrease. Based on chain length, C14 increases, and C15-containing CE species appear following treatment (Figure 10b and Figure 13b). Concurrently, in the SM class, DB = 0 increases, DB = 1 decreases and C32 chain length decreases (Figure 10a and Figure 13a). This specific profile of the changes elucidates the “cholesterol paradox,” which is one of the primary metabolic contradictions caused by AS1411 described in the literature. Multi-omics analyses indicate that AS1411 downregulates rate-limiting enzymes in the cholesterol biosynthesis pathway (HMGCR, SQLE, FDFT1) at both the mRNA and protein levels; yet, total intracellular cholesterol levels do not decrease and instead show an increase per cell [56]. The experimental CE dataset provides an explanation for this paradox: under AS1411 stress, rather than leaving free cholesterol in cellular membranes, the cancer cell converts it into cholesteryl esters (specifically DB = 0, C14, and C15 forms) via the Sterol O-Acyltransferase 1 (SOAT1/ACAT1) enzyme, sequestering it within cytoplasmic lipid droplets. This sequestration of free cholesterol suppresses cholesterol sensors (the SCAP/SREBP complex) in the endoplasmic reticulum (ER) membranes, triggering a feedback mechanism that transcriptionally downregulates HMGCR and SQLE genes despite high total cellular cholesterol [69]. Additionally, the increase in DB = 0 SM exerts a notable effect on membrane biophysics. The cell membrane contains specialized microdomains termed “lipid rafts,” which are formed by the assembly of cholesterol and fully saturated sphingolipids [58]. Lipid rafts are rigid and thick platforms relative to surrounding membrane regions, facilitating receptor clustering and signal transduction [70]. Measurements using Di-4-ANEPPS ratiometric probes demonstrate that while AS1411 treatment does not alter plasma membrane fluidity, it decreases the fluidity of intracellular organelle membranes [56]. The observed increase in fully saturated (DB = 0) SM and specific CE accumulation provides biochemical evidence for the formation of saturated lipid microdomains in endosomal and ER membranes, which rigidifies the membranes and decreases fluidity.

### 4.2. Analysis of Cellular Stress and Death Pathways at the Lipid Sub-Species Level

Transitioning from general lipid classes to molecularly defined lipid sub-species reveals the specific components through which the AS1411 aptamer alters the metabolic wiring of cancer cells. This sub-species resolution helps identify the direct targets of cell death mechanisms, signaling cascades, and cellular stress responses in cancer.

#### 4.2.1. Neutral Lipid Sub-Species DAG and TAG and the Methuotic Vacuolization Response

Neutral lipids, namely DAG and TAG play critical roles in energy storage, signal transduction, and protection against membrane lipotoxicity [71]. Analysis of sub-species data reveals that in the DAG class, species 30:0;0, 32:0;0, 34:0;0, and 36:3;0 are upregulated, species 30:1;0, 33:0;0, 36:0;0, and 38:6;0 are synthesized following sequence-specific treatment, whereas common signaling DAG species such as 32:1;0, 33:1;0, 34:1;0, 34:2;0, 36:2;0, 36:4;0, and 38:4;0 are downregulated (Figure 14a and Figure 17a). DAG molecules serve as second messengers in the activation of the Protein Kinase C (PKC) pathway, particularly at the plasma membrane. The appearance of a highly unsaturated, long-chain, and bulky molecule like DAG 38:6;0 indicates that the cancer cell, under AS1411-induced stress, may modulate specific membrane curvature or coordinate the inward collapse of macropinosomes [61]. The TAG class represents the biochemical reservoir most shifted by AS1411 application. In the dataset, a total of 19 large TAG species—including 42:0;0, 44:1;0, 47:0;0, 48:2;0, 49:0;0, 49:1;0, 50:3;0, 51:1;0, 52:5;0, 54:0;0, 54:6;0, 56:4;0, 56:5;0, 56:6;0, 56:7;0, 58:1;0, 58:4;0, 58:5;0, and 58:6;0—appear in the treated groups. The literature indicates that AS1411, through a NCL-dependent mechanism, hyperactivates the Rac1 protein, triggering a process that drives massive fluid and membrane internal consumption (macropinocytosis), eventually leading to cytoplasmic vacuolization and cell lysis (methuosis) [63]. The enlargement of macropinosomes induces an accumulation of fluid and membrane in the cytoplasm [61]. To compensate for this expanded surface area and prevent free fatty acid lipotoxicity arising from internal cellular membranes, the cancer cell upregulates its TAG synthesis machinery. The appearance of TAGs containing highly PUFAs (e.g., 56:7;0 and 58:6;0) indicates a cellular response to remove reactive unsaturated lipids from organelle zars (ER, mitochondria, lysosomes) and sequester them within the inert cores of neutral lipid droplets [65].

#### 4.2.2. Rearrangement of Phospholipid Sub-Species (PE, PC, PG, PI, PS) and Ferroptozis

When examining the PE sub-species profile, 36:4;0, 38:4;0, 38:6;0, and 40:5;0 species are found to be upregulated. Conversely, species such as 32:1;0, 34:1;0, 36:2;0, and 40:7;0 are downregulated (Figure 15b and Figure 18b). It is established that arachidonic acid- or adrenic acid-bound PEs, such as PE 36:4 and PE 38:4, function as primary initiating substrates for ferroptosis. These lipids are synthesized via the ACSL4 enzyme and peroxidized by the lipoxygenase (ALOX) family, resulting in irreversible cell membrane permeabilization [66]. The specific expansion of the PE 36:4 and PE 38:4 pool in cancer cells following AS1411 stimulation establishes a biochemical framework for lipid peroxidation. The synergistic anti-tumor efficacy observed in recent studies combining AS1411 aptamer nanocarriers with erianin, sorafenib, or other ferroptosis inducers can be attributed to AS1411 directly priming the cell for ferroptosis by upregulating these specific PE sub-species [67]. In the PC class, the 40:7;0 molecule appears in the treated groups, while 36:2;0 is downregulated, and 28:0;0 and 34:4;0 are upregulated (Figure 15a and Figure 18a). The incorporation of a large, highly unsaturated PC molecule (40:7;0) into the plasma membrane can induce structural instability within fluidity microdomains. This indicates that cell-surface NCL internalization alters plasma membrane lipid dynamics. In the PI class, 40:4;0 and 40:5;0 species are upregulated and 34:2;0, 36:2;0, 36:3;0, and 38:5;0 species are downregulated, while 34:0;0 is detected following AS1411 treatment (Figure 15d and Figure 18d). For macropinosomes to traffic, shrink, and fuse with lysosomes, specific phosphoinositide signaling cascades dependent on the PI backbone (such as the conversion of PI(3)P to PI(3,5)P2 via PIKFYVE kinases) must function correctly [61]. The upregulation of long-chain/highly unsaturated PI sub-species like 40:4 and 40:5 implies that AS1411 alters the macropinosome–lysosome fusion mechanism at the phospholipid level, contributing to vacuole entrapment and the development of methuosis pathology [61]. Similarly, the appearance of 37:1;0 and 38:5;0 in the PS class (Figure 15e and Figure 18e), and 30:0;0, 36:0;0, and 42:8;0 in the PG class (Figure 15c and Figure 18c), indicates modifications in plasma membrane asymmetry and mitochondrial inner membranes [68].

#### 4.2.3. SM and CE Sub-Species on Lipid Rafts

Within the SM class, 32:1;2 and 34:1;2 are downregulated, 34:0;2 is upregulated, while 36:0;2 and 42:0;2 are synthesized following treatment (Figure 16a and Figure 19a). Simultaneously, in the CE class, 14:0;1, 15:0;0, and 17:0;0 species emerge, and 14:0;0 and 18:0;0 species are upregulated, 16:1;0 and 18:1;0 are downregulated (Figure 16b and Figure 19b). These two sets of findings correlate within the context of the “Lipid Rafts” theory in cancer cell biology [72]. Lipid rafts are hydrophobic micro-platforms formed by cholesterol and sphingolipids that cluster signaling receptors (e.g., EGFR, Fas/CD95, Nucleolin) and possess lower fluidity [58]. NCL bound to the AS1411 aptamer depends on lipid rafts for internalization or interaction with secondary cofactors at the plasma membrane. The synthesis of SM species containing fully saturated and long ceramide backbones (34:0;2, 36:0;2, 42:0;2; Figure 16a and Figure 19a), paired with the accumulation of short-chain CEs (14:0;0, 14:0;1, 15:0;0, and 17:0;0, and 18:0;0; Figure 16b and Figure 19b), indicates that AS1411 expands and mechanically rigidifies the lipid raft regions of intracellular membranes (particularly endosomes, the ER, and the trans-Golgi network). This provides a molecular basis for the literature finding that AS1411 decreases intracellular membrane fluidity without affecting the plasma membrane in NIH-3T3 cells, as measured by Di-4-ANEPPS ratiometric analyses [56]. While the cell diverts PUFAs toward lipid droplets and PEs, it accumulates saturated sphingomyelins within the endomembrane system, reducing internal membrane fluidity.

To evaluate the translational significance of the observed lipidomic changes in a broader therapeutic context, it is essential to consider the clinical history of AS1411. Chen et al. (2007) conducted a comprehensive mechanistic study to evaluate the pharmacological efficacy and therapeutic spectrum of nucleolin (NCL)-targeting in an acute myeloid leukemia (AML) model [73]. The primary objective of their study was to evaluate the selective sensitivity, internalization dynamics, and downstream apoptotic pathways triggered by AS1411 in the MV4-11 AML cell line, alongside patient-derived primary AML blasts. Their findings demonstrated that these AML models exhibit rapid, NCL-mediated endocytosis of AS1411, leading to a potent, micromolar-range cytotoxic and anti-proliferative response that was entirely absent in the inactive control oligonucleotide (CRO) groups. At the molecular level, Chen et al. revealed that upon internalization, AS1411 successfully disrupts the protective interaction of cytosolic NCL with anti-apoptotic transcripts, directly inducing Bcl-2 mRNA destabilization and subsequent caspase-mediated apoptosis in MV4-11 cells and primary blasts. While this specific leukemia model outlines a classic, direct apoptotic cascade via oncogenic mRNA degradation, it provides a compelling contrast to the specialized lipidome remodeling observed in our U-87 glioblastoma cells. This divergence suggests that while the NCL-driven entry mechanism of AS1411 remains universally conserved across distinct malignancies, the ultimate metabolic or structural execution pathways are heavily dictated by the unique homeostatic requirements of the specific tumor lineage. As a pioneering NCL-targeted DNA aptamer, AS1411 has successfully progressed to Phase II clinical trials for the treatment of acute myeloid leukemia (AML) [74,75]. The primary objective of these studies were to compare the response rates, safety, and tolerability between patients receiving cytarabine alone versus those treated with a combination of AS1411 and cytarabine. By demonstrating the clinical feasibility, safety, and therapeutic activity of AS1411 in combination with chemotherapy, these randomized Phase II trials provide a strong translational foundation for targeting NCL in aggressive malignancies. Rosenberg et al. [39] conducted a single-arm Phase II clinical trial evaluating the therapeutic activity and safety of AS1411 in patients with metastatic, clear-cell, renal cell carcinoma (RCC) who had failed treatment with one or more prior tyrosine kinase inhibitors. Although the unselected cohort showed minimal overall activity (2.9% response rate), one patient exhibited a dramatic and durable 84% reduction in tumor burden. Whole exome sequencing of this responder identified missense mutations in mTOR and FGFR2, highlighting a therapeutic link to NCL’s physiological role in upregulating AKT1/mTOR signaling pathways. While these clinical trials underscore the safety and systemic feasibility of NCL-targeted therapies, they focus on therapeutic endpoints driven by rapid blast clearance (in AML) or the inhibition of canonical intracellular signaling cascades (in RCC). Given that NCL overexpression on the plasma membrane and its subsequent endocytosis upon AS1411 binding represent a generalized, non-cell-type-specific entry mechanism, the initiating biophysical change in the cell membrane is expected to share fundamental commonalities across different cancer lineages. However, the downstream metabolic consequences of this targeted intervention diverge significantly in glioblastoma. Unlike blood cancers or renal carcinomas, glioblastoma exhibits an extreme dependence on lipid scavenging, *de novo* lipogenesis, and cholesterol homeostasis to sustain its rapid proliferation and invasive capability.

Consequently, the sequence-specific, bidirectional lipidome remodeling observed in U-87 cells in the present study unveils a specialized metabolic vulnerability that may not be as critical in AML or RCC. Rather than merely inducing non-specific cell death, NCL inhibition directly target the physical and metabolic infrastructure of the glioblastoma membrane. These findings suggest that in lipid-dependent brain tumors, the therapeutic bottleneck is metabolic rather than purely signaling-driven. By disrupting these precise lipid networks and introducing structural membrane stress, AS1411 treatment metabolically primes these highly aggressive brain tumor cells, potentially lowering the threshold required to achieve clinical efficacy and synergistic responses when combined with secondary targeted therapies, standard apoptotic signaling, and/or ferroptotic interventions.

Malignant tumors like glioblastoma are often characterized by incomplete differentiation, and differentiation therapy has shown promise in this context. Investigation into post-treatment changes in lipid profiles is essential for evaluating therapeutic efficacy and understanding the relationship between treatment effects and lipidomic alterations. The effects of differentiation-inducing agents, sodium phenylbutyrate (SPB) and ATRA, on U87-MG cells were previously explored in a study by Liu et al. MALDI-MS was utilized to profile the membrane lipidome of U-87 glioblastoma cells undergoing differentiation therapy induced by sodium phenylbutyrate (SPB) and all-trans retinoic acid (ATRA) [76]. SPB, a histone deacetylase inhibitor, has demonstrated some potential for remission in recurrent glioblastoma, while ATRA, a vitamin A metabolite, is known to influence cell growth and apoptosis. Under those differentiation conditions, Liu et al. reported increases in monounsaturated phosphatidylcholine (PC) species—specifically PC 38:1, *m*/*z* 816; PC 36:1, *m*/*z* 788; and PC 31:1, *m*/*z* 725, alongside a concurrent reduction in the saturated species PC 32:0 *m*/*z* 734. Our results revealed different dynamics for these particular species. Specifically, PC 36:1 and PC 31:1 were found to increase under AS1411 treatment compared to the untreated control (Figure 15a). Furthermore, the saturated PC 32:0 species, which Liu et al. reported to decrease under differentiation therapy, actually exhibited a significant increase following AS1411 exposure in comparison to the control (Figure 15a). However, when evaluated alongside the control sequence, no statistically significant divergence was noted for PC 36:1, PC 31:1, or PC 32:0 levels between the AS1411 and CRO treatment cohorts. Because the CRO treatment also prompted comparable alterations without a statistical distinction between the two aptamers, these specific shifts cannot be confidently evaluated as AS1411-specific or NCL-dependent therapeutic modulations. Instead, they likely represent a non-specific background response to macromolecular oligonucleotide delivery. Additionally, while Liu et al. highlighted a marked increase in PC 38:1 under SPB and ATRA exposure, this species was not identified as significantly altered in the current study. Conversely, this study identified a distinct subset of PC sub-species that were altered in an AS1411-specific manner—none of which were reported to change in the study by Liu et al. Specifically, the exclusive emergence of the highly polyunsaturated species PC 40:7 was observed solely in the AS1411 group, along with a sequence-specific upregulation of PC 28:0 and PC 34:4. Additionally, a targeted downregulation of PC 36:2 was observed following AS1411 exposure Figure 18a). These variations in the PC profiles point to a potential difference in the underlying mechanisms of action. The SPB and ATRA treatments analyzed by Liu et al. stimulate a classical differentiation program that gradually adapts the plasma membrane toward a physiologically stable state. In contrast, the exclusive accumulation of the highly polyunsaturated PC 40:7 and the targeted upregulation of short-/medium-chain species (PC 28:0 and PC 34:4) observed under AS1411 treatment may reflect an NCL-targeted cellular stress response. Rather than inducing a standard differentiation pathway, AS1411 treatment appears to prompt a notable homeostatic disruption in U-87 cells, characterized by a specific membrane remodeling process that could potentially render the cells more susceptible to lipotoxic stress and associated therapeutic vulnerabilities.

The effects of gamma-linolenic acid (GLA) and ionizing radiation on the lipidome of U87 MG glioblastoma cells were previously investigated by Antal et al. (2015) [77]. Specifically, Antal et al. found that GLA incubation leads to a coordinated upregulation of critical phospholipid species containing 3 or 4 double bonds, driven by direct enzymatic elongation into DGLA and subsequent incorporation into PC 36:3, 36:4, 38:3 and 38:4, PE 38:4, PI 38:3, and PS 38:3.. Conversely, our lipidomics data in the current study revealed a highly selective and distinct regulatory pattern for these exact species under NCL-targeted treatment. Within the PC and PI pools, species such as PC 36:3,36:4, and 38:3, and PI 38:3 (Figure 15a and Figure 18d), exhibited only non-specific alterations, showing no statistically significant divergence between the AS1411 and CRO groups. On the other hand, while Antal et al. highlighted significant increases in PS 38:3 and PC 38:4 species, these specific sub-species were not detected within the AS1411-treated U-87 cell lipidome. Furthermore the downregulation of PC 36:2 and the exclusive emergence of PC 40:7 under AS1411 treatment, were not observed in the study conducted by Antal et al.

Lipidomic changes in U-87 cells treated with adenoviruses (therapeutic Ad-p53 or DI312 control) or the cytotoxic chemotherapeutic agent (SN-38) were previously investigated by Görke et al. [78]. In their polar lipid fraction analysis, significant changes in hydroxylated PI lipid species were observed only when adenovirus was combined with SN-38, culminating in a 100-fold increase in PI (38:6-O). Under those apoptotic conditions, Görke et al. reported significant increases in the abundance of some PI and PG classes, particularly highlighting the upregulation of hydroxylated species such as PI (38:6-O) only when adenovirus was combined with SN-38, and the hydroxylated PG species (32:0-O) and (32:1-O) was observed only in the Ad-p53/SN38 treatment group. The present study revealed different and highly nuanced dynamics for these particular classes. While Görke et al. observed a prominent upregulation of hydroxylated PI and PG species during p53-mediated apoptosis, these oxidized/hydroxylated sub-species were not identified as significantly altered or accumulated in the current study. This indicates that direct lipid hydroxylation may not be a primary driver of the immediate membrane remodeling associated with AS1411 exposure under these specific experimental conditions. Conversely, the platform utilized in this study identified a distinct subset of PI and PG sub-species that were altered in a strictly AS1411-specific manner—none of which were reported to change in the study by Görke et al. Specifically, exclusive emergence of PI 34:0 (Figure 18d) as well as PG 30:0, 36:0, and 42:8 appeared solely in the AS1411 group (Figure 15c and Figure 18c). Furthermore, a sequence-specific upregulation of PI 40:4 and 40:5 (Figure 18d), along with a targeted downregulation of PG 40:5 (Figure 18c) and PI 34:2, 36:2, 36:3, and 38:5 (Figure 18d), was uniquely observed in following AS1411 exposure, with no comparable alterations found in the CRO cohort. These variations in the PI and PG profiles suggest a potential difference in the underlying mechanisms of action. The p53 gene therapy and chemotherapy combination analyzed by Görke et al. triggers a classical, transcriptionally driven apoptotic program that heavily involves mitochondrial permeabilization and associated reactive oxygen species (ROS) generation, likely explaining the accumulation of hydroxylated PI and PG species. In contrast, the exclusive accumulation of specific structural species such as PI 34:0 and PG 30:0, 36:0, and 42:8 observed under AS1411 treatment may reflect a NCL-targeted cellular stress response. These contrasting results likely reflect the radically different mechanisms of action between direct NCL-targeting by AS1411 and standard adenovirus/chemotherapy combinations, highlighting distinct pathways affecting critical PI and PG signaling axes.

### 4.3. Limitations of the Study

The U-87 cells remain the universally accepted gold standard in the neuro-oncology literature for mapping lipid metabolic reprogramming, nucleolin overexpression dynamics, and cell-fate decisions under aptamer therapeutic stress. As this represents the first systematic study in the literature investigating the direct and uncoupled effects of AS1411 on the U-87 glioblastoma cell lipidome, our research strategy presents vertical depth (delving into unprecedented lipid molecular detail within a single, thoroughly benchmarked cellular system) over horizontal breadth (superficial profiling across multiple cell lines). To achieve this level of depth, we utilized a high-resolution shotgun lipidomics platform with an high number of biological replicates (*n* = 5 per group; at least three out of five biological replicates are reported), allowing us to quantitatively map hundreds of precise molecular lipid species and their sub-species transitions with rigorous reproducibility. Additionally, employing advanced analytical methodologies like liquid chromatography-mass spectrometry (LC-MS) would enhance the detection and quantification limits for minor signaling lipid components. While the absence of multiple cell lines is an inherent limitation, the massive molecular dataset generated within this single standardized model provides a robust, reproducible, and statistically rigorous foundation.

## 5. Conclusions

The cytotoxic effects and comprehensive lipidomic alterations induced by the nucleolin-targeting aptamer AS1411 in U-87 glioblastoma cells were investigated in this study. This comprehensive analysis demonstrates that the therapeutic and cytotoxic effect of the AS1411 nucleolin aptamer on cancer cells is not restricted to antiproliferative gene silencing or simple signaling pathway inhibition. Instead, it constitutes a systemic and disruptive metabolic rewiring of the cancer cell’s structural framework—its membrane lipidome. Synthesizing the global class, saturation, and carbon chain length profiles with the specific sub-species profiles alongside existing literature yields a multi-layered biochemical model of how AS1411 dictates cellular fate. At the core of this process lies the high-affinity interaction between AS1411 and the NCL protein, which is upregulated on the surface and within the cytoplasm of cancer cells. Upon activation of this interaction, the cell initiates an internal consumption loop of fluid and membrane—hyperstimulated macropinocytosis—potentially as a defense response or due to altered signaling (such as Rac1 hyperactivation). However, the internalized large macropinosomes fail to fuse with lysosomes for degradation due to altered signaling associated with shifted PI sub-species (e.g., the upregulation of PI 40:4 and PI 40:5, and the post-treatment appearance of PI 34:0). Instead, they coalesce and expand within the cell. This non-apoptotic cell death process, termed methuosis, demands high rates of new membrane and lipid synthesis. The synthesis of 19 specific, unsaturated, and bulky TAG forms ranging from 42:0;0 to 58:6;0 reflects the cell’s biosynthetic response to this pathological membrane demand, serving as an attempt to sequester reactive free PUFA lipotoxicity into non-membrane compartments like lipid droplets. Crucially, the data reveal how AS1411 bridges different cell death pathways. Although AS1411 suppresses cholesterol biosynthesis at the transcriptomic level (HMGCR, SQLE downregulation), literature indicates that total cellular cholesterol does not drop. This cholesterol paradox is clarified by the experimental data showing the upregulation of CE 14:0;0 and 18:0;0 alongside the appearance of CE 14:0;1, 15:0;0, and 17:0;0 sub-species: the cell rapidly converts available free cholesterol into CE via ACAT1/SOAT1 enzymes for storage. The withdrawal of free cholesterol, combined with the shift in SM 34:0;2, 36:0;2, and 42:0;2 toward fully saturated (DB = 0) forms, increases the density of lipid raft microdomains in the ER and endosomes. This biophysical accumulation provides a molecular basis for the reduction in intracellular membrane fluidity observed via Di-4-ANEPPS ratiometric analyses. During this metabolic stress and vacuolization, AS1411 introduces a secondary therapeutic vulnerability within the cellular lipidome: a ferroptotic priming state. The detected enrichment of PE is characterized by a mass accumulation of specific arachidonic and adrenic acid-containing PE 36:4;0, 38:4;0, 38:6;0, and 40:5;0 species under the DB = 4, 5, 6 profile. The incorporation of these primary targets of iron-dependent, ALOX-mediated lipid peroxidation into cell membranes indicates that the aptamer’s mechanism sensitizes the cancer cell to ferroptotic stimuli. This molecular priming aligns with literature findings where combining AS1411 conjugates with erianin yields enhanced anti-tumor elimination.

Ultimately, our comprehensive lipidomic analysis reveals that alongside inducing methuotic vacuolization stress, AS1411 critically diminishes intracellular membrane fluidity, thereby predisposing the cancer cells to ferroptotic cell death. These specific lipid alterations not only provide critical mechanistic insights into the aptamer’s anti-tumor activity but also highlight potential lipid-based vulnerabilities in glioblastoma that could be exploited for diagnostic or therapeutic purposes.

## Data Availability

The raw lipidomic mass spectrometry datasets in this study are available from Lipotype GmbH under the quote number QS-24-1036-1.

## Supplementary Materials

The following supporting information can be downloaded at https://www.mdpi.com/article/10.3390/biom16081091/s1, Figure S1: Cell viability assessed by MTT assay following 48 h treatment with varying concentrations of AS1411 and CRO against U-87 cells. Data represent mean ± SD (*n* = 5). CRO:Control aptamer; Figure S2: Cell viability assessed by MTT assay following 96 h treatment with varying concentrations of AS1411 and CRO against U-87 cells. Data represent mean ± SD (*n* = 5). CRO:Control aptamer, Figure S3: Variation in amounts of lipids measured in quality control reference samples (*n* = 2 ± SD). Table S1: Experimental Header. Overview of analyzed sample cohorts including control (C1–C5), CRO (CRO1–CRO5), and treated sample groups (Apt1–Apt5) along with their corresponding short names and full names. Table S2: Analyzed Sample Quantities. Summary of the volume (µL) of each sample analyzed across control, CRO, and sample cohorts. Table S3: Coefficients of Variation (CV) for Reference Samples. Median CV percentages calculated across different lipid classes (CE, DAG, PA, PC, PE, PI, PS, SM, TAG) to demonstrate analytical reproducibility and quality control. Table S4: Lipid Annotation and Structural Database. Comprehensive structural classification, functional category, shorthand notations, and corresponding SwissLipids identifiers and names for all identified lipid features. Table S5: Raw Data (mol%_Raw) for Double Bond Count and Chain Length Distributions. Raw dataset detailing the combined double bond (DB) numbers and carbon chain length profiles across all analyzed samples. Table S6: Absolute Quantification Data in Picomoles (pmol_Raw). Raw quantitative concentrations (expressed in pmol) of identified lipid sub-species across all individual samples. Table S7: Relative Abundance Data (mol%_Raw). Raw relative abundance (expressed in mol%) of identified lipid sub-species across all individual samples. Table S8: Lipid Saturation Remodeling Summaries (Double Bonds, Figure 8, Figure 9 and Figure 10). Categorized summary of lipid double bond counts, observed saturation remodeling patterns, and AS1411 sequence-specificity associated with Figure 8, Figure 9 and Figure 10. Table S9: Lipid Chain Length Remodeling Summaries (Figure 11, Figure 12 and Figure 13). Detailed profile of carbon chain length modulation, observed remodeling effects, and AS1411 sequence-specificity across lipid classes corresponding to Figure 11, Figure 12 and Figure 13. Table S10: Sub-species Level Remodeling in Absolute Amounts (pmol, Figure 14, Figure 15 and Figure 16). Summary of sub-species level lipid alterations evaluated in absolute pmol concentrations and AS1411 sequence-specificity linked to Figure 14, Figure 15 and Figure 16. Table S11: Sub-species Level Remodeling in Relative Amounts (mol%, Figure 17, Figure 18 and Figure 19). Summary of sub-species level lipid alterations evaluated in molar percentages (mol%) and AS1411 sequence-specificity linked to Figure 17, Figure 18 and Figure 19.
