# Supplementary material for: AS1411-Induced Lipidomic Alterations and Therapeutic Insights in U-87 Glioblastoma Cells
Source: Biomolecules. 2026 Jul 25;16(8):1091. doi: 10.3390/biom16081091 (PMC13510112; doi:10.3390/biom16081091)
Supplement: Supplementary file 1 [file biomolecules-16-01091-s001.zip › Supplementary Figures.pdf]

AS1411-Induced Lipidomic Alterations and Therapeutic Insights in U-87 Glioblastoma Cells

Ozan Kılıçkaya [1,*] and Serap Şahin [2,*]

[1] Department of Pharmaceutical Biotechnology, Faculty of Pharmacy, Afyonkarahisar Health Sciences University, 03030 Afyonkarahisar, Türkiye
[2] Department of Biochemistry, Faculty of Pharmacy, Afyonkarahisar Health Sciences University, 03030 Afyonkarahisar, Türkiye
* Correspondence: ozan.kilickaya@afsu.edu.tr (O.K.); serap.sahin@afsu.edu.tr (S.Ş.); Tel.: +90-5423472844 (O.K.); +90-5336294893 (S.Ş.)

Supplementary Figures

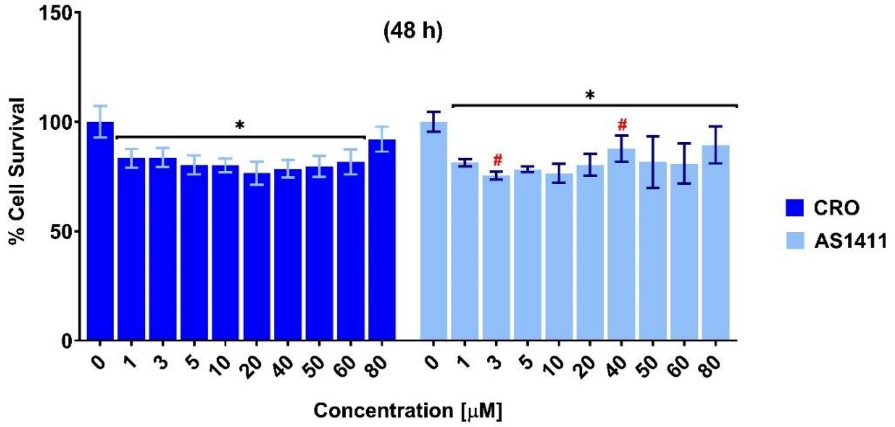

Figure S1. Cell viability assessed by MTT assay following 48 h treatment with varying concentrations of AS1411 and CRO against U-87 cells. Data represent mean ± SD (n=5). Statistical significance: * $p < 0.05$ vs. Control; # $p < 0.05$ vs. CRO. CRO:Control aptamer.

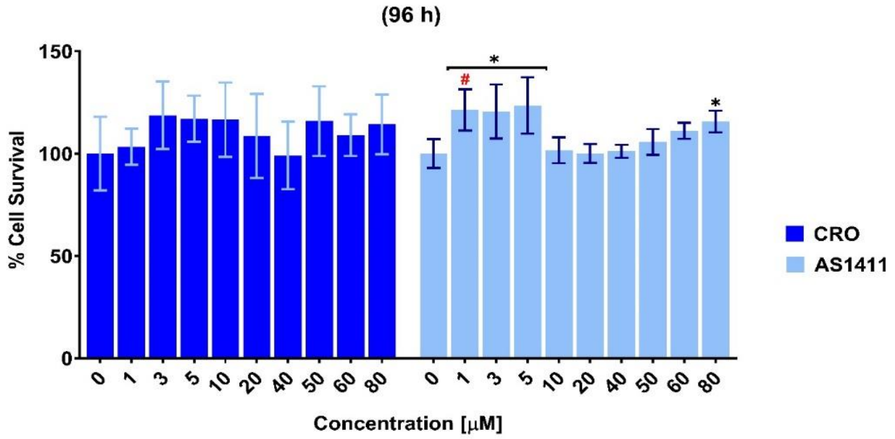

Figure S2. Cell viability assessed by MTT assay following 96 h treatment with varying concentrations of AS1411 and CRO against U-87 cells. Data represent mean ± SD (n=5). Statistical significance: * $p < 0.05$ vs. Control; # $p < 0.05$ vs. CRO. CRO:Control aptamer.

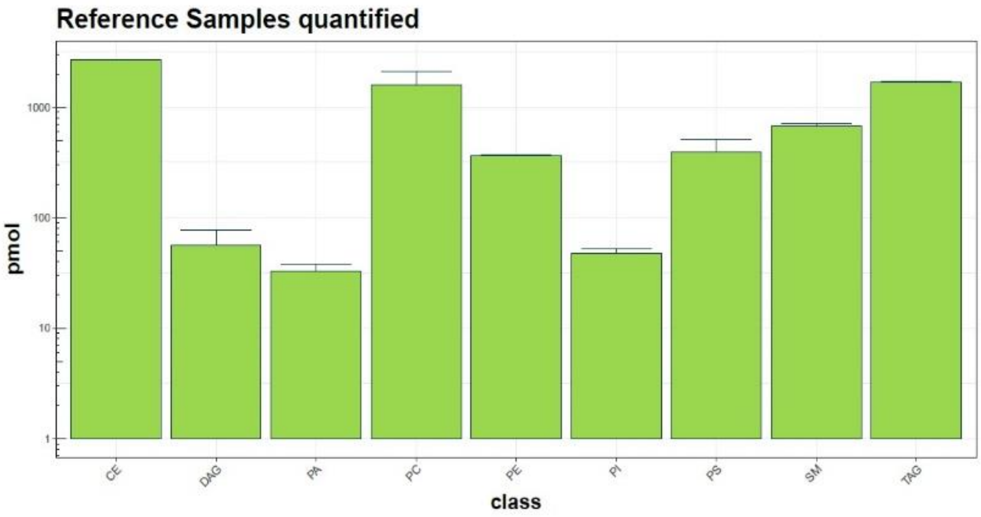

FigureS3. Variation in amounts of lipids measured in quality control reference samples (n= 2 ± SD).
